# Viral RNA-binding ability conferred by SUMOylation at PB1 K612 of influenza A virus is essential for viral pathogenesis and transmission

**DOI:** 10.1371/journal.ppat.1009336

**Published:** 2021-02-11

**Authors:** Junping Li, Libin Liang, Li Jiang, Qian Wang, Xia Wen, Yuhui Zhao, Pengfei Cui, Yaping Zhang, Guangwen Wang, Qibing Li, Guohua Deng, Jianzhong Shi, Guobin Tian, Xianying Zeng, Yongping Jiang, Liling Liu, Hualan Chen, Chengjun Li

**Affiliations:** State Key Laboratory of Veterinary Biotechnology, Harbin Veterinary Research Institute, Chinese Academy of Agricultural Sciences, Harbin, The People’s Republic of China; The Ohio State University, UNITED STATES

## Abstract

Posttranslational modifications, such as SUMOylation, play specific roles in the life cycle of invading pathogens. However, the effect of SUMOylation on the adaptation, pathogenesis, and transmission of influenza A virus (IAV) remains largely unknown. Here, we found that a conserved lysine residue at position 612 (K612) of the polymerase basic protein 1 (PB1) of IAV is a bona fide SUMOylation site. SUMOylation of PB1 at K612 had no effect on the stability or cellular localization of PB1, but was critical for viral ribonucleoprotein (vRNP) complex activity and virus replication in vitro. When tested in vivo, we found that the virulence of SUMOylation-defective PB1/K612R mutant IAVs was highly attenuated in mice. Moreover, the airborne transmission of a 2009 pandemic H1N1 PB1/K612R mutant virus was impaired in ferrets, resulting in reversion to wild-type PB1 K612. Mechanistically, SUMOylation at K612 was essential for PB1 to act as the enzymatic core of the viral polymerase by preserving its ability to bind viral RNA. Our study reveals an essential role for PB1 K612 SUMOylation in the pathogenesis and transmission of IAVs, which can be targeted for the design of anti-influenza therapies.

## Author summary

IAV has evolved to exploit the host posttranslational modifications system for its own benefit. The transcription and replication of IAV genome occur in the nucleus of infected cells, which is catalyzed by the RNA-dependent RNA polymerase (RdRp). PB1 is the catalytic subunit and the assembly core of the RdRp. The ability to efficiently bind viral RNA by PB1 is a prerequisite for the RdRp to fulfil its function. In this study, we demonstrated that PB1 protein from different subtypes of IAV is a target of SUMOylation in both transfected and infected cells, and identified K612 of PB1 as the key SUMOylation site. The vRNP complex activity, replication in vitro, pathogenicity in mice and airborne transmission among ferrets were dramatically attenuated when the SUMOylation-defective PB1/K612R mutation was introduced. Notably, we found that SUMOylation at K612 is essential for PB1 to acquire the ability to efficiently bind viral RNA, thus allowing for the RdRp to transcribe and replicate the viral genome. Our findings therefore thoroughly explore the contribution of PB1 SUMOylation on influenza infection and establish SUMOylation site PB1 K612 as a potential target for anti-influenza drug development.

## Introduction

Influenza A virus (IAV) is an important zoonotic pathogen that causes frequent epidemics and occasional pandemics in humans. Based on the antigenicity of the surface glycoproteins hemagglutinin (HA) and neuraminidase (NA), IAVs are classified into 18 different HA subtypes and 11 different NA subtypes [1]. Since 1900, humans have suffered four influenza pandemics, among which the most recent 2009 pandemic H1N1 virus transmitted to over 215 countries and territories between April 2009 and August 2010 [2]. Moreover, humans are also constantly facing threats posed by avian influenza viruses (AIVs), with H5N1 and H7N9 human infections as two prime examples. The first human infection with H5N1 AIV occurred in Hong Kong in 1997 [3], and between 2003 and 2020, 861 human infection cases were reported, of which 455 were fatal [4]. The H7N9 low pathogenic AIVs identified in 2013 and the subsequently mutated H7N9 highly pathogenic AIVs in 2017 [5–8] led to 1568 human infections, including 615 fatal cases [9]. Therefore, continued efforts to elucidate the factors and mechanisms underlying the pathogenesis and transmission of IAVs remain critical for the development of novel anti-influenza therapies.

The viral ribonucleoprotein (vRNP) complex of IAVs is composed of individual viral RNA segments, three polymerase subunits [i.e., polymerase basic protein 1 (PB1), polymerase basic protein 2 (PB2), polymerase acidic protein (PA)], and nucleoprotein (NP) [10]. PB1 functions as the catalytic subunit and the assembly core of the RdRp [11–13], which is responsible for the transcription and replication of the IAV genome [14,15]. In addition to its N- and C-terminal extensions that make contact with PA and PB2, respectively, PB1 contains a central region comprising finger, fingertips, palm, and thumb domains [16], which are characteristics of RdRp. Terminal initiation during replication is dependent on the priming loop in the thumb domain of PB1 [17], which is also essential for transcriptional elongation, but not for transcriptional initiation [17,18].

SUMOylation is an important regulatory posttranslational protein modification mechanism in eukaryotic cells. SUMO (small ubiquitin-related modifier) is a ubiquitin-like protein that is covalently conjugated to a target protein. There are four SUMO forms in humans, SUMO1, SUMO2, SUMO3, and SUMO4, among which SUMO1-SUMO3 are ubiquitously expressed and covalently conjugated to the lysine residues of target proteins [19]. SUMOs are conjugated to target proteins via a three-step cascade, involving a heterodimeric E1 activating enzyme, SAE1/SAE2; a unique E2 conjugating enzyme, Ubc9; and several E3 ligases [19,20]. The SUMOylation of a particular substrate may interfere with or facilitate its interaction with binding partners, regulate its activity, function, subcellular localization, and can affect its stability [21,22].

IAV proteins are also targets of SUMOylation. To date, three IAV proteins [i.e., non-structural protein 1 (NS1), matrix protein 1 (M1), and NP] have been clearly identified as substrates of SUMO and the role of their SUMOylation in virus replication has been clarified. Among them, NS1 was the first identified SUMOylated IAV protein, the SUMOylation of which leads to enhanced protein stability and improved virus growth in cell culture [23,24]. SUMOylation of M1 has been shown to facilitate the nuclear export of vRNP complexes and the assembly of virus particles [25]; SUMOylation of NP is essential for the intracellular trafficking of NP and for virus growth in vitro [26]. More IAV proteins could be targets of SUMO modification, and IAV infection has been shown to induce a global increase in cellular SUMOylation [27,28]. However, the role of SUMO modification in the pathogenesis and transmission of IAV has not been explored.

Here, we demonstrated that PB1, the catalytic core of the viral RdRp, is SUMOylated by SUMO1 in both transfected and virus-infected cells. We found a highly conserved lysine residue at position 612 (K612) is the key SUMO acceptor site of PB1. The SUMOylation of PB1 at K612 was found to be critical for vRNP complex activity and virus replication in vitro. Mechanistically, the SUMOylation of PB1 at K612 was essential for PB1 to function in the binding of vRNA. Importantly, a SUMOylation-deficient PB1 K612R mutation significantly attenuated the virulence of H1N1, H5N1 and H7N9 viruses in mice. Furthermore, the replication and respiratory droplet transmission of the PB1 K612R mutant of a 2009 pandemic H1N1 virus in ferrets was reduced compared with the wild-type virus, which created a stress condition that drove the R612K reversion mutation in inoculated and exposed ferrets.

## Results

### IAV PB1 is mainly SUMOylated by SUMO1

**Fig 1:**
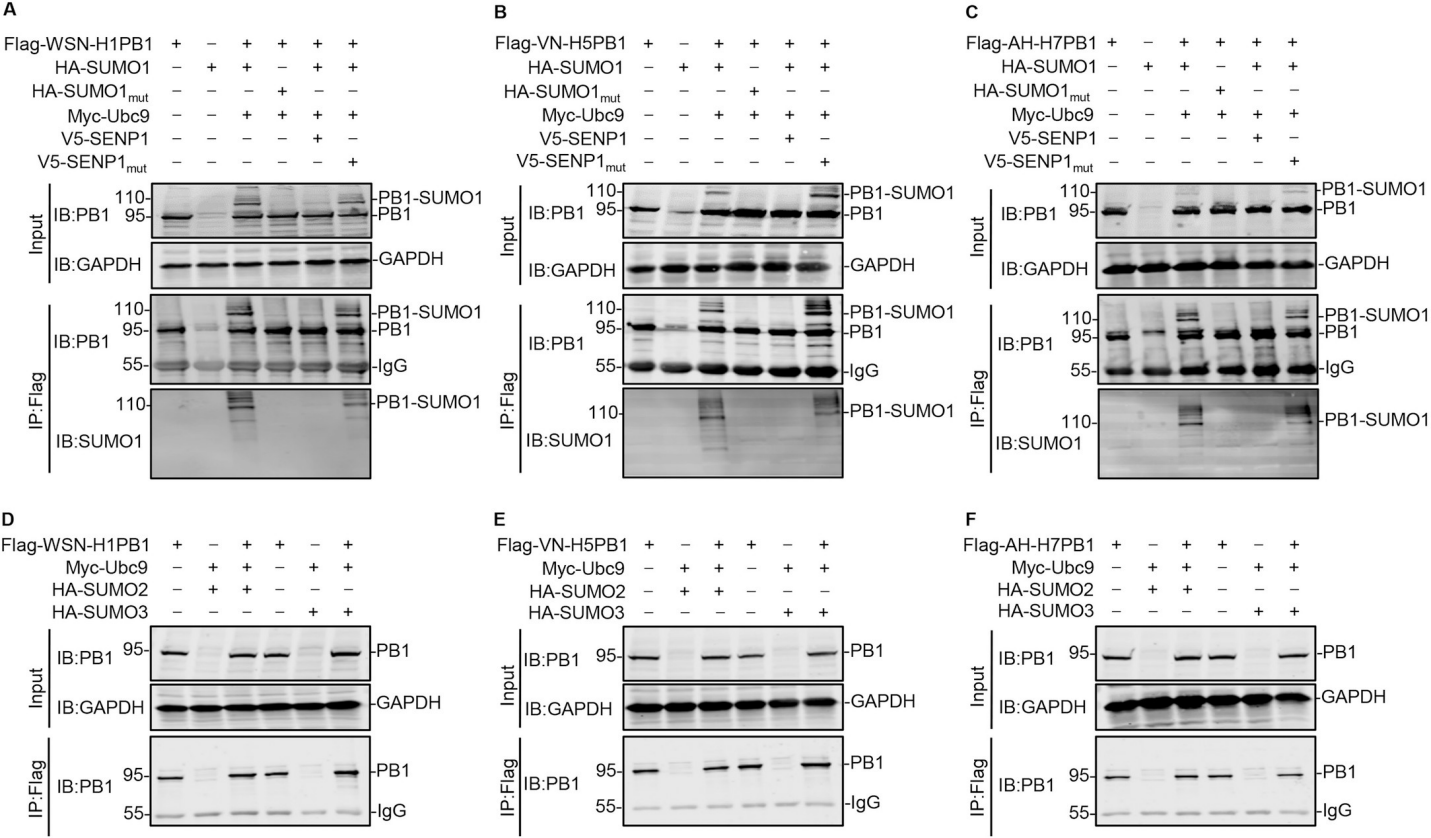
IAV PB1 is mainly SUMOylated by SUMO1. (A-C) HEK293T cells were transfected with plasmids expressing Flag-tagged PB1 from WSN (H1N1) (A), VN/1180 (H5N1) (B), or AH/1 (H7N9) (C), together with or without SUMO1 or SUMO1_mut_, Ubc9, and SENP1 or SENP1_mut_. At 48 h post-transfection, cell lysates were prepared, immunoprecipitated with anti-Flag M2 agarose beads, and western blotted to detect PB1 and SUMO1 with the indicated antibodies. (D-F) HEK293T cells were transfected with plasmids encoding Flag-tagged PB1 from WSN (H1N1) (D), VN/1180 (H5N1) (E), or AH/1 (H7N9) (F), together with or without Ubc9, and SUMO2 or SUMO3. At 48 h post-transfection, cell lysates were prepared, immunoprecipitated with anti-Flag M2 agarose beads, and western blotted to detect PB1 with an anti-PB1 mAb. The values on the left of the blots are molecular sizes in kilodaltons of the corresponding bands. Data shown are representative of three independent experiments.

Given the importance of SUMOylation in the posttranslational modification of viral proteins, we first investigated whether IAV PB1, the core subunit of viral RdRp, is a target of the host SUMOylation system. HEK293T cells were cotransfected with Flag-tagged PB1 of A/WSN/33 (WSN, H1N1) virus (WSN-H1PB1), along with or without SUMO-conjugating enzyme Ubc9, SUMO1 or SUMO1_mut_ (a conjugation-deficient SUMO1 variant), and SUMO-specific protease SENP1 or SENP1_mut_ (SENP1 with loss-of-function mutations) [24,26]. At 48 h post-transfection, the cell lysates were immunoprecipitated with anti-Flag agarose, and then western blotted with an anti-PB1 monoclonal antibody (mAb). When WSN-H1PB1 was cotransfected with Ubc9 and SUMO1, a clear additional band for PB1 of ~110 kDa was detected, reflecting the potential modification of PB1 (95 kDa) by SUMO1. In contrast, this 110-kDa band was absent from cells cotransfected with SUMO1_mut_, indicating that WSN-H1PB1 is indeed SUMOylated (Fig 1A). To confirm these results, we introduced an expression construct for the de-SUMOylating enzyme SENP1 or SENP1_mut_ into the transfection. The high molecular weight band of SUMOylated PB1 at ~110 kDa was markedly reduced when cotransfected with SENP1, whereas it was unaffected in the SENP1_mut_-expressing cells (Fig 1A). Of note, the same molecular weight bands of WSN-H1PB1 were also detected when the precipitated proteins were analyzed by using an anti-SUMO1 mAb, further confirming that WSN-H1PB1 was modified by SUMO1 (Fig 1A).

Next, we examined the SUMO1 modification of PB1 of other IAV subtypes and found that the PB1 proteins of A/chicken/Vietnam-Ca Mau/1180/2006 (VN/1180, H5N1) (VN-H5PB1) and A/Anhui/1/2013 (AH/1, H7N9) (AH-H7PB1) were also SUMOylated by SUMO1 in transfected HEK293T cells (Fig 1B and 1C). Collectively, these results indicate that SUMOylation of IAV PB1 by SUMO1 is a common posttranslational modification that lacks viral subtype specificity.

Finally, we determined whether IAV PB1 was also SUMOylated by the other two forms of SUMO, SUMO2 and SUMO3. As shown in Fig 1D–1F, WSN-H1PB1, VN-H5PB1, and AH-H7PB1 were not SUMOylated when cotransfected with Ubc9 and SUMO2 or SUMO3.

Taken together, these results clearly demonstrate that IAV PB1 protein is a target for SUMOylation by SUMO1, but not SUMO2 or SUMO3.

### The lysine residue at position 612 (K612) is the SUMOylation site of IAV PB1

**Fig 2:**
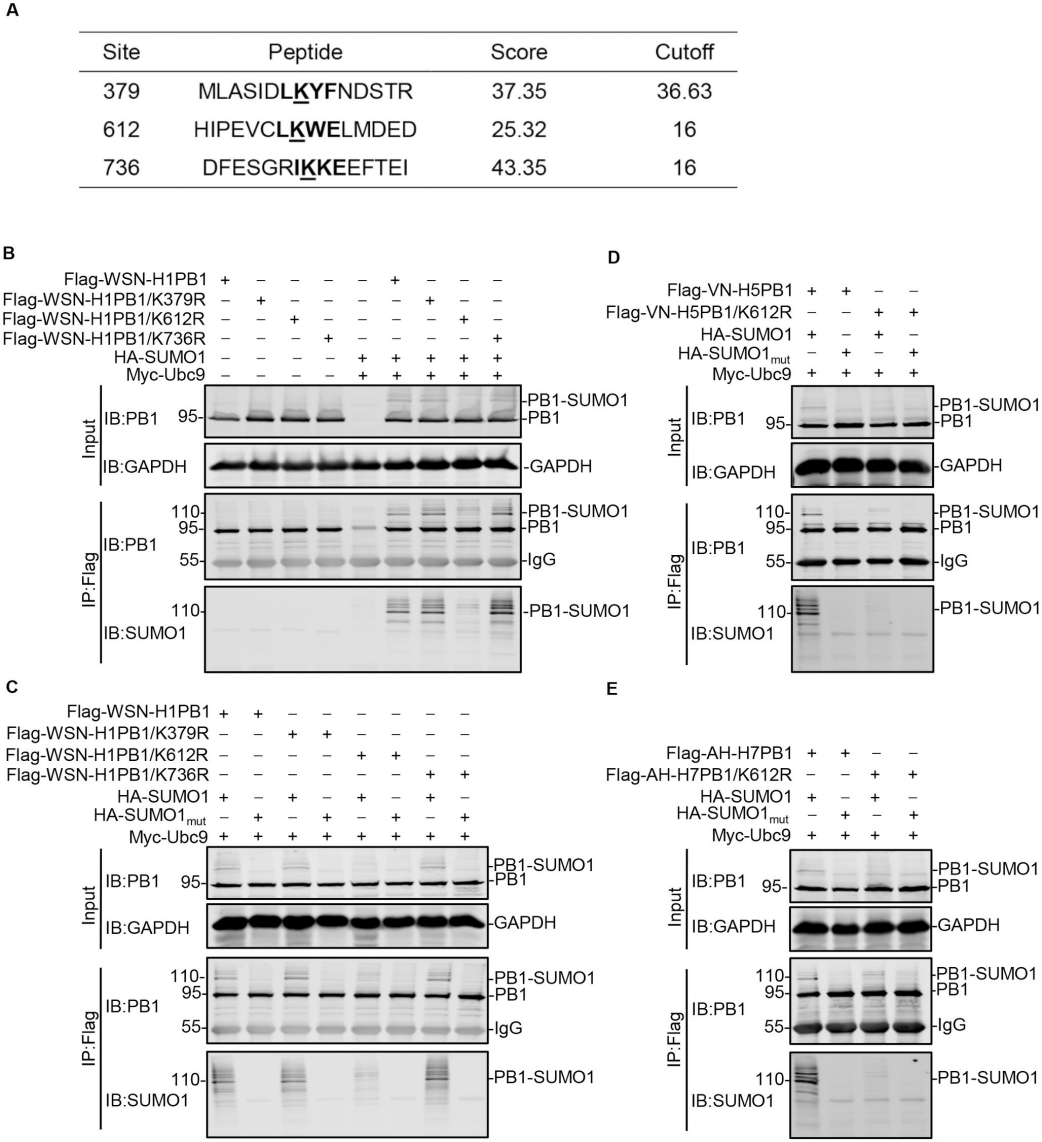
K612 is the key SUMOylation site of IAV PB1. (A) Candidate SUMOylated sites (indicated in boldface and underline) in IAV PB1 were predicted by using the GPS-SUMO software. (B) HEK293T cells were transfected with plasmids encoding Flag-tagged WSN-H1PB1 or its potential SUMOylation site mutant WSN-H1PB1/K379R, WSN-H1PB1/K612R, or WSN-H1PB1/K736R, together with or without SUMO1 and Ubc9. Forty-eight hours later, cell lysates were prepared, immunoprecipitated with anti-Flag M2 agarose beads, and western blotted with an anti-PB1 or anti-SUMO1 mAb. (C) HEK293T cells were transfected with plasmids encoding Flag-tagged WSN-H1PB1 or the indicated potential SUMOylation site mutant, together with SUMO1 or SUMO1_mut_, and Ubc9. Immunoprecipitation (IP) and western blotting were performed as in (B). (D, E) HEK293T cells were transfected with plasmids encoding Flag-tagged VN/1180-H5PB1 (D) or AH/1-H7PB1 (E), or their corresponding K612R mutant, together with SUMO1 or SUMO1_mut_, and Ubc9. IP and western blotting were performed as in (B). Data shown are representative of three independent experiments in (B-E).

To identify the SUMOylation site(s) of PB1 protein, we used the in silico prediction program GPS-SUMO [29]. Three lysine residues, K379, K612, and K736, were predicted to be potential candidate sites for SUMO-conjugation in WSN-H1PB1 (Fig 2A). Individual K-to-R mutant PB1 proteins for all three predicted lysine residues were then generated, and their effect on the SUMOylation of PB1 was examined by coimmunoprecipitation (co-IP) and western blotting analysis. The precipitated proteins were detected by using an anti-PB1 mAb and an anti-SUMO1 mAb, respectively. As expected, WSN-H1PB1 was SUMOylated to form a 110-kDa high molecular weight band when cotransfected with Ubc9 and SUMO1. The main SUMOylated band was also present in the lanes transfected with the PB1/K379R and PB1/K736R mutants, but not the PB1/K612R mutant. These results indicate that K612, but not K379 or K736, is the key SUMO acceptor site of WSN-H1PB1 (Fig 2B).

To confirm that K612 was indeed the major SUMO acceptor site, the WSN-H1PB1 and WSN-H1PB1 mutants were each cotransfected with Ubc9 and SUMO1 or SUMO1_mut_, and then co-IPed and western blotted. As shown in Fig 2C, the 110-kDa PB1-SUMO1 band was detected when WSN-H1PB1, WSN-H1PB1/K379R, or WSN-H1PB1/K736R was cotransfected with SUMO1, whereas the indicated SUMOylated PB1 band disappeared when SUMO1_mut_ was used instead of SUMO1. However, when the WSN-H1PB1/K612R mutant was cotransfected with SUMO1 or SUMO1_mut_, the 110-kDa PB1-SUMO1 band was not detected. These data further confirm that K612 is the authentic SUMOylation site of WSN-H1PB1.

We next determined whether VN-H5PB1 and AH-H7PB1, which both possess K612, were also SUMOylated at this site. Compared with wild-type VN-H5PB1 and AH-H7PB1, which were SUMOylated by SUMO1, analysis of the VN-H5PB1/K612R and AH-H7PB1/K612R mutants did not reveal the 110-kDa PB1-SUMO1 band (Fig 2D and 2E), indicating that the lysine residue at position 612 is also a major SUMOylation site of VN-H5PB1 and AH-H7PB1. By performing a systemic sequence analysis involving 14,226 PB1 sequences, we found that the K612 residue is highly conserved among different IAV subtypes (S1 Table). Collectively, our results indicate that the K612 residue is a common SUMO acceptor site of the IAV PB1 proteins.

### PB1 is SUMOylated at K612 in infected cells

**Fig 3:**
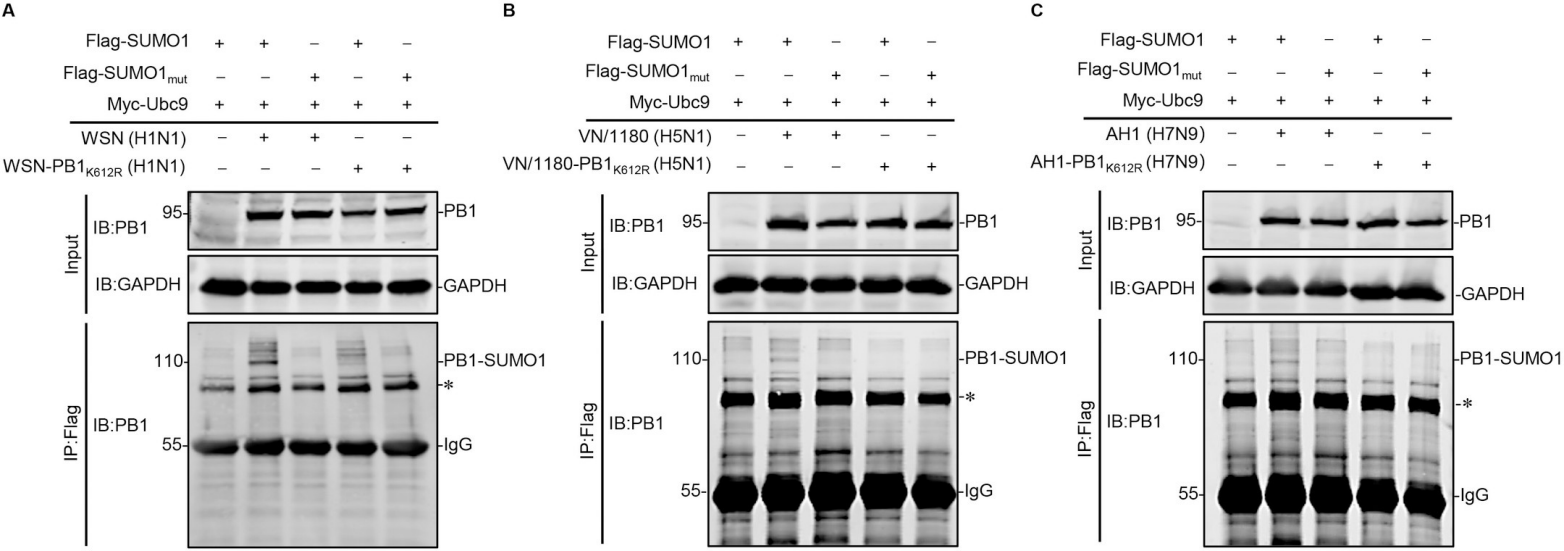
IAV PB1 is SUMOylated in infected cells. HEK293T cells were transfected with plasmids encoding Flag-tagged SUMO1 or SUMO1_mut_, and Ubc9. At 24 h post-transfection, the cells were infected with WSN (H1N1) or WSN-PB1_K612R_ (H1N1) (A), VN/1180 (H5N1) or VN/1180-PB1_K612R_ (H5N1) (B), and AH/1 (H7N9) or AH/1-PB1_K612R_ (H7N9) (C) at an MOI of 2. Eighteen hours later, the cell lysates were immunoprecipitated with anti-Flag M2 agarose beads and western blotted with an anti-PB1 mAb. The asterisk indicates unknown protein. Data shown are representative of three independent experiments.

To address whether the SUMOylation of PB1 protein occurs during IAV infection, SUMOylation-defective PB1/K612R mutants of WSN (H1N1), VN/1180 (H5N1), and AH/1 (H7N9) viruses (WSN-PB1_K612R_, VN/1180-PB1_K612R_, and AH/1-PB1_K612R_) were generated by reverse genetics and deep-sequenced to ensure that they contained no unwanted mutations (S2 Table). HEK293T cells transiently overexpressing Ubc9 and SUMO1-Flag or SUMO1_mut_-Flag were infected with WSN (H1N1), VN/1180 (H5N1), AH/1 (H7N9), or their PB1/K612R mutants at an MOI of 2. Eighteen hours later, cell lysates were immunoprecipitated with anti-Flag agarose and then western blotted with an anti-PB1 mAb. As shown in Fig 3A, a band of 110 kDa corresponding to PB1-SUMO1 was detected in WSN (H1N1) virus-infected cells transfected with SUMO1 and Ubc9; whereas the indicated PB1-SUMO1 band disappeared when SUMO1_mut_ was used in place of SUMO1. In contrast, the 110-kDa PB1-SUMO1 band was not detectable in cells infected with the WSN-PB1_K612R_ (H1N1) virus (Fig 3A). When tested in the background of the VN/1180 (H5N1) and AH/1 (H7N9) viruses, similar results were obtained (Fig 3B and 3C). These data demonstrate that IAV PB1 is SUMOylated at K612 in infected cells.

### SUMOylation at K612 does not affect the stability or cellular localization of PB1

**Fig 4:**
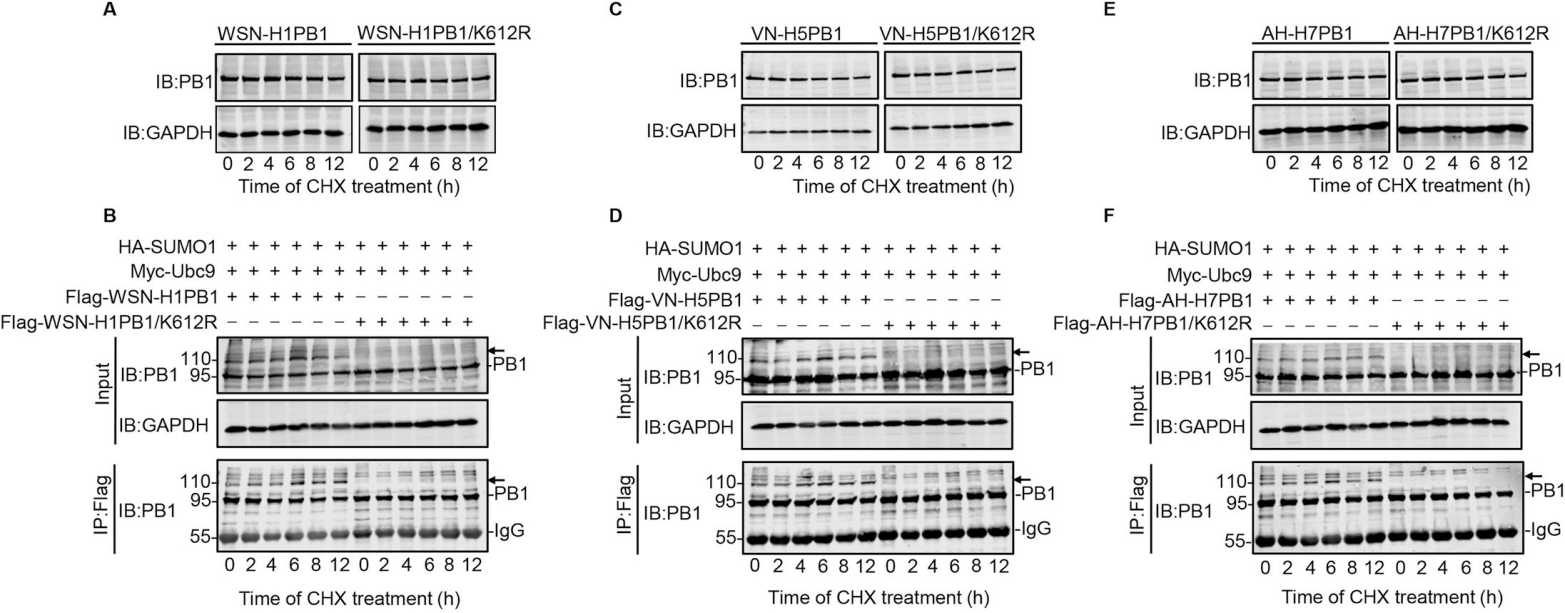
SUMOylation at K612 does not affect the stability of IAV PB1. (A, C, and E) HEK293T cells were transfected with plasmids encoding WSN-H1PB1 or WSN-H1PB1/K612R (A), VN/1180-H5PB1 or VN/1180-H5PB1/K612R (C), or AH/1-H7PB1 or AH/1-H7PB1/K612R (E). At 24 h post-transfection, the cells were treated with 100 μg/ml cycloheximide (CHX). The steady-state levels of PB1 protein after CHX treatment were then assessed at the indicated time points by western blotting with an anti-PB1 mAb. Western blots of whole cell lysates with an anti-GAPDH mAb are shown as loading controls. (B, D, and F) HEK293T cells were transfected with plasmids encoding Flag-tagged WSN-H1PB1 or WSN-H1PB1/K612R (B), VN/1180-H5PB1 or VN1180-H5PB1/K612R (D), or AH/1-H7PB1 or AH/1-H7PB1/K612R (F), together with SUMO1 and Ubc9. At 24 h post-transfection, the cells were treated with 100 μg/ml CHX for the time indicated. The cell lysates were then immunoprecipitated with anti-Flag M2 agarose beads, and both the precipitated proteins and the whole cell lysates were western blotted by using an anti-PB1 mAb. Arrow, SUMOylated PB1 protein. Data shown are representative of three independent experiments.

After identifying K612 as the major SUMOylation site of IAV PB1, we investigated whether the stability of PB1 is affected by SUMOylation at K612. HEK293T cells were transfected with an expression plasmid encoding WSN-H1PB1 or WSN-H1PB1/K612R mutant. At 24 h post-transfection, protein synthesis was blocked with cycloheximide (CHX) and the steady-state levels of PB1 after CHX treatment were monitored by use of western blotting. Both PB1 and PB1/K612R protein levels remained stable at 0, 2, 4, 6, 8, and 12 h under CHX treatment (Fig 4A), indicating that SUMOylation at K612 has no effect on the stability of IAV PB1. To further demonstrate that SUMOylation of PB1 at K612 does not alter protein stability, we cotransfected Flag-tagged WSN-H1PB1 or WSN-H1PB1/K612R mutant with Ubc9 and SUMO1 in HEK293T cells. As shown in Fig 4B, both native PB1 and SUMOylated PB1 were present in cells expressing WSN-H1PB1, and the level of total PB1 remained stable across different time points; native PB1, but not the 110-kDa PB1-SUMO1, was detected in cells expressing WSN-H1PB1/K612R, whose level remained unchanged. These results demonstrate that the stability of IAV PB1 is unaffected by the SUMOylation status of the K612 residue. We also found that SUMOylation at K612 did not affect the stability of VN-H5PB1 or AH-H7PB1 (Fig 4C–4F). Together, these data show that SUMOylation at K612 does not affect the stability of IAV PB1.

**Fig 5:**
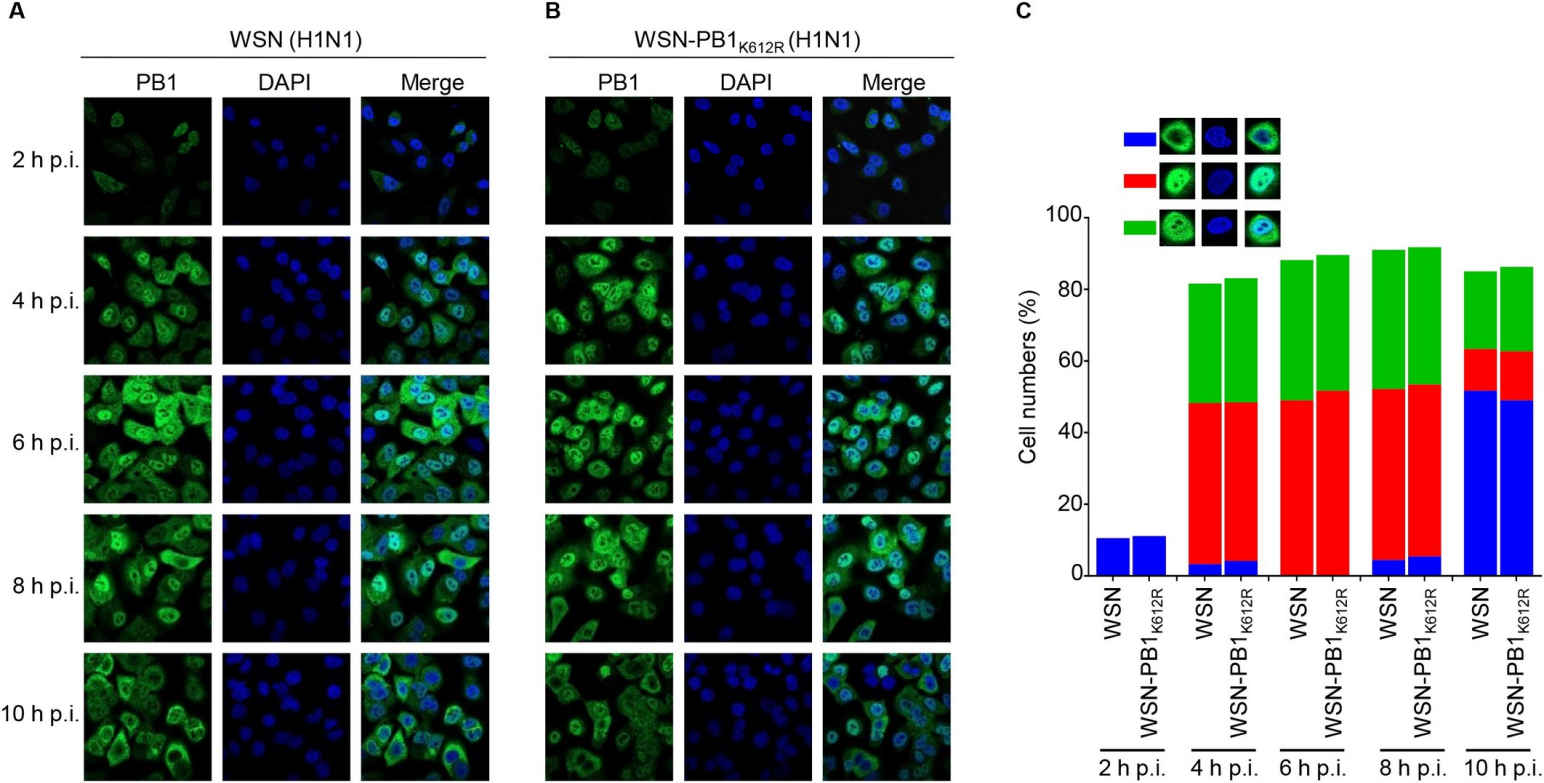
SUMOylation at K612 does not affect the cellular localization of PB1. (A, B) A549 cells were infected with WSN (H1N1) (A) or WSN-PB1_K612R_ (H1N1) (B) at an MOI of 5, and the cells were fixed at the indicated time points, followed by staining with a rabbit anti-PB1 pAb and incubation with Alexa Fluor 488 donkey anti-rabbit IgG (H+L). The nuclei were stained with DAPI. Representative images are shown. (C) Quantitative analysis of PB1 localization in virus-infected cells. The results shown are calculated from one hundred cells. On the basis of the confocal microscopy in panels A and B, the localization of PB1 was categorized into three types: predominantly cytoplasmic localization (blue), clear nuclear localization (red), and simultaneous localization in the cytoplasm and nucleus (green).

Since SUMOylation could potentially affect the cellular localization of target proteins, we examined whether SUMOylation at K612 altered the intracellular distribution of IAV PB1. A549 cells were infected with WSN (H1N1) or WSN-PB1_K612R_ (H1N1) virus at an MOI of 5, and the cellular localization of the PB1 protein was analyzed by use of an immunofluorescence assay. As shown in Fig 5A and 5B, at 2 h post-infection (p.i.), the fluorescence signal of PB1 was very weak, and PB1 mainly localized to the cytoplasm of both WSN- and WSN-PB1_K612R_-infected cells. At 4 h p.i., PB1 had entered the nucleus and appeared to accumulate there. At 6 h and 8 h p.i., the PB1 signal was strong in the nucleus of the infected cells, and by 10 h p.i., PB1 was largely exported from the nucleus and had accumulated in the cytoplasm. Overall, no observable quantitative difference was found between WSN- and WSN-PB1_K612R_-infected cells with respect to the localization of PB1 across the infection course (Fig 5C). These results indicate that PB1 SUMOylation at 612K does not influence the cellular localization of PB1 during virus infection.

### SUMOylation at PB1 K612 does not affect the assembly of the viral RNP complex

**Fig 6:**
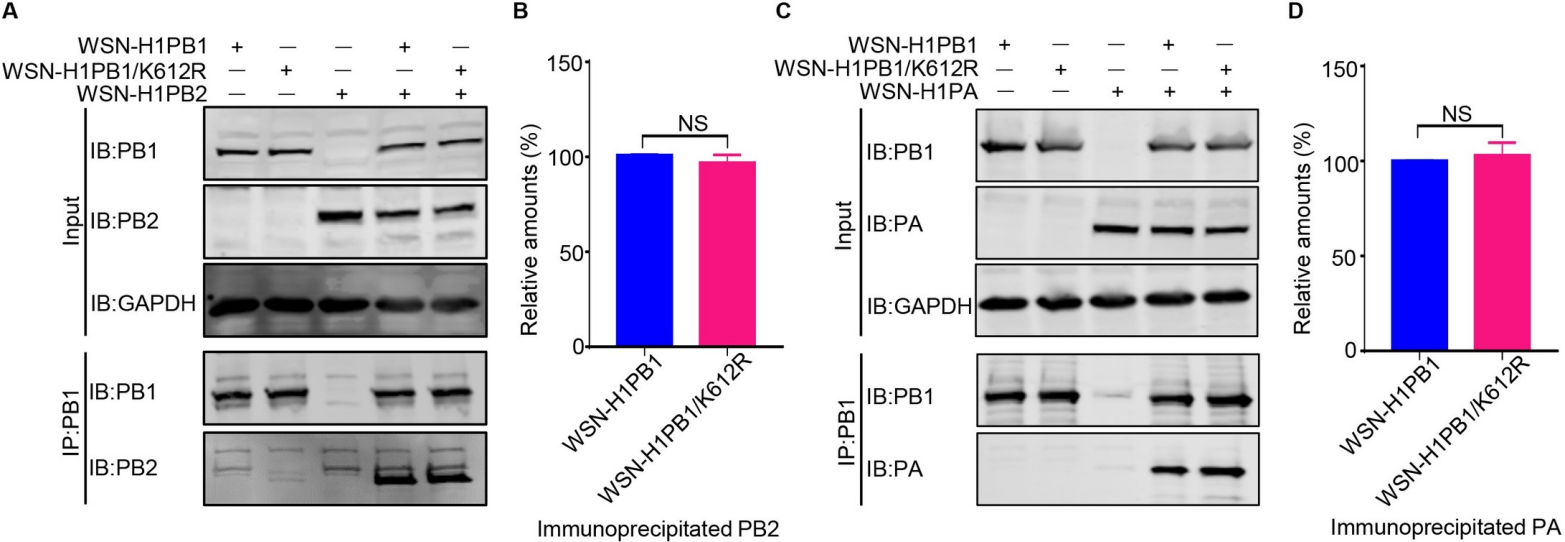
SUMOylation at K612 of PB1 has no effect on the interaction of PB1 with PB2 or PA. (A) PB1/K612R mutation does not affect the PB1-PB2 interaction. HEK293T cells were transfected individually or in combination as indicated with plasmids expressing WSN-H1PB1 or WSN-H1PB1/K612R mutant, and WSN-H1PB2. Forty-eight hours later, cell lysates were immunoprecipitated with a mouse anti-PB1 mAb, followed by western blotting with antibodies against PB1 and PB2. (B) Quantification of immunoprecipitated PB2 in (A). Band intensities of western blots from three assays were quantified by using ImageJ software, and the amount of precipitated PB2 was normalized to that of precipitated PB1. (C) PB1/K612R mutation does not affect the PB1-PA interaction. HEK293T cells were transfected individually or in combination as indicated with plasmids expressing WSN-H1PB1 or WSN-H1PB1/K612R mutant, and WSN-H1PA. Forty-eight hours later, cell lysates were immunoprecipitated with a mouse anti-PB1 mAb, followed by western blotting with antibodies against PB1 and PA. (D) Quantification of immunoprecipitated PA in (C) as described in (B). The amount of precipitated PA was normalized to that of precipitated PB1. The results are expressed as the mean ± SD of three assays and the significance was tested with a Student’s t-test (B, D). NS, not significant.

PB1 is the core subunit of the viral heterotrimeric polymerase complex, with its N-terminal region interacting with PA and its C-terminal region binding PB2 [12,13]. We therefore tested whether the SUMOylation of PB1 at K612 affects the PB1-PA and PB1-PB2 interactions. HEK293T cells were transfected for 48 h with WSN-H1PB1 or WSN-H1PB1/K612R mutant individually or simultaneously were transfected with WSN-H1PB2 or WSN-H1PA. Cell lysates were immunoprecipitated with anti-PB1 mAb and then western blotted to detect PB1, PB2, or PA. The amount of PB2 or PA that was immunoprecipitated by WSN-H1PB1 or WSN-H1PB1/K612R was comparable (Fig 6), indicating that the interactions between PB1-PB2 and PB1-PA were not affected by the SUMOylation at K612 of PB1. We also tested whether the SUMOylation at PB1 K612 affects the assembly of the viral RNP complex. Viral RNP complexes, bearing WSN-H1PB1 or WSN-H1PB1/K612R, were reconstituted in HEK293T cells by co-transfection of expression plasmids for PB2, PB1 or PB1/K612R, PA, and NP of WSN (H1N1) virus, together with pHH21-SC09NS F-Luc, which was used to provide a vRNA-like template RNA [30–32]. At 48 h post-transfection, the vRNP complexes were immunoprecipitated from cell lysates by using an anti-PB1 mAb, and the presence of PB2, PA, and NP in the precipitated vRNP complexes was detected by western blotting. No significant differences in the amounts of PB2, PA, and NP were observed between vRNP complexes bearing WSN-H1PB1 and those bearing WSN-H1PB1/K612R (S1 Fig). These results indicate that SUMOylation at K612 of PB1 has no effect on the assembly of the vRNP complex of IAV.

### SUMOylation at K612 of PB1 enhances vRNP complex activity

**Fig 7:**
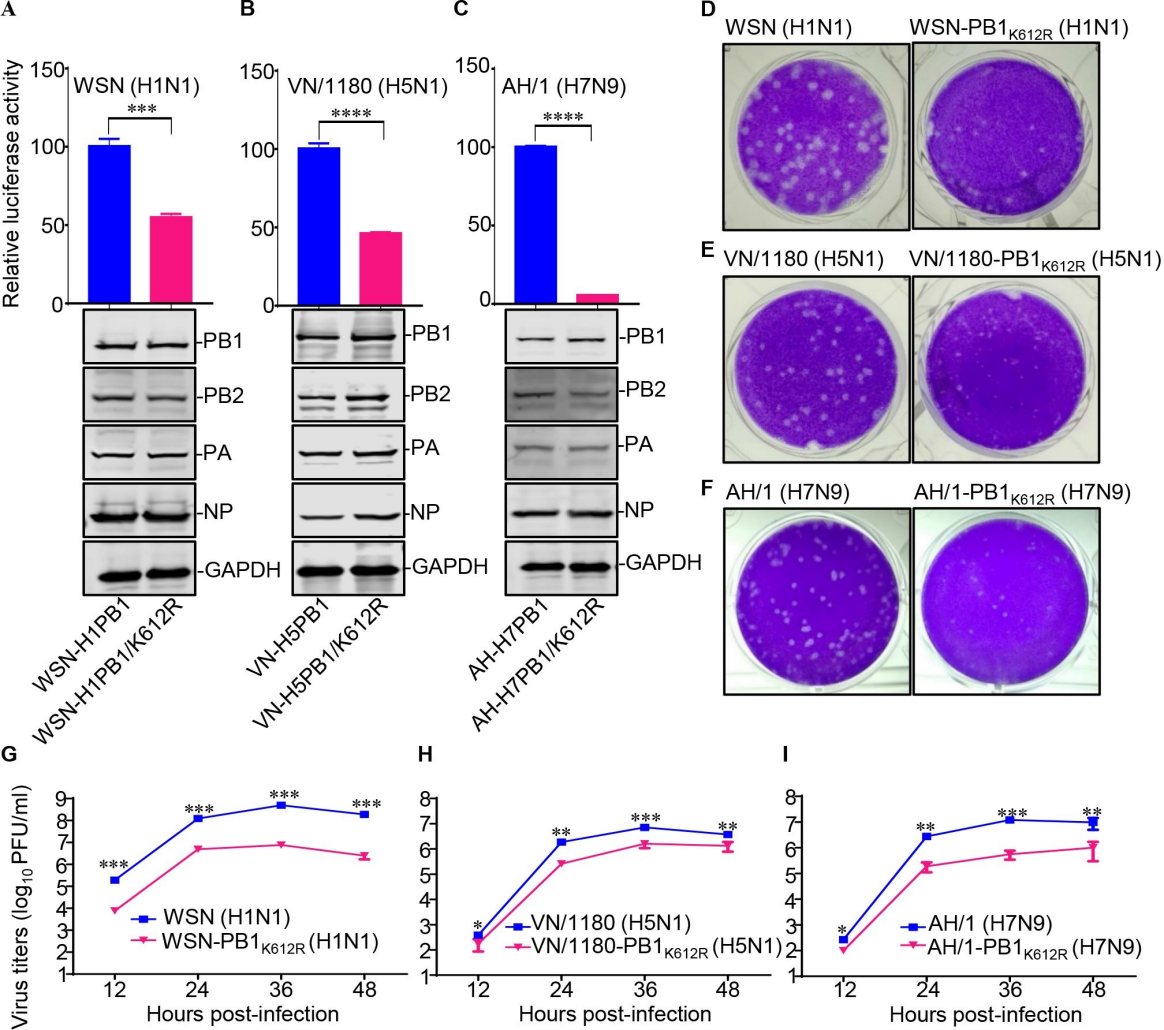
SUMOylation at K612 of IAV PB1 is essential for vRNP complex activity and virus replication in vitro. (A-C) Four vRNP protein expression plasmids (PB2, PA, NP, and PB1 or a PB1/K612R mutant) derived from WSN (H1N1) (A), VN/1180 (H5N1) (B), or AH/1 (H7N9) (C), together with a luciferase reporter, pHH21-SC09NS F-Luc, and an internal control reporter, pRL-TK, were cotransfected into HEK293T cells. Forty-eight hours later, the cell lysates were subjected to a dual-luciferase reporter assay. Western blotting was also performed to assess the expression of the transfected vRNP protein constructs (bottom panel). (D-F) Plaque phenotypes of WSN (H1N1) or WSN-PB1_K612R_ (H1N1) (D), VN/1180 (H5N1) or VN/1180-PB1_K612R_ (H5N1) (E), and AH/1 (H7N9) or AH/1-PB1_K612R_ (H7N9) (F) on MDCK cells. (G-I) Growth curves of WSN (H1N1) or WSN-PB1_K612R_ (H1N1) (G), VN/1180 (H5N1) or VN/1180-PB1_K612R_ (H5N1) (H), and AH/1 (H7N9) or AH/1-PB1_K612R_ (H7N9) (I). MDCK cells were inoculated with the indicated viruses at an MOI of 0.001, and the supernatants were collected at the indicated time points to determine the virus titers by use of plaque assays. Data shown are representative of three independent experiments. The results are expressed as the mean ± standard deviation (SD) of triplicates and the significance was tested with a Student’s t-test (A-C) or a one-way ANOVA followed by t-test (G-I). *, *P* < 0.05; **, *P* < 0.01; ***, *P* < 0.001; ****, *P* < 0.0001.

The vRNP complex of IAV is responsible for the transcription and replication of the viral genome [14,15]. We therefore asked whether vRNP complex activity was affected by SUMOylation of PB1 at K612. To this end, a minigenome assay was performed in HEK293T cells, which were transfected with the four vRNP complex protein expression plasmids, PB2, PB1 or PB1/K612R, PA, and NP of WSN (H1N1), VN/1180 (H5N1), or AH/1 (H7N9) virus, together with a reporter plasmid, pHH21-SC09NS F-Luc, encoding a vRNA-like firefly luciferase gene [30–32]. Forty-eight hours later, the luciferase activity of the cell lysates was measured to reveal the vRNP complex activity. The results showed that the vRNP complex activity of WSN (H1N1), VN/1180 (H5N1), and AH/1 (H7N9) virus decreased by approximately 50%, 55%, and 95%, respectively, in cells expressing PB1/K612R compared with that of PB1 (Fig 7A–7C). These findings demonstrate that the SUMOylation of PB1 at K612 is essential for the vRNP complex activity of IAV.

### SUMOylation-defective PB1/K612R mutation impairs IAV replication

To determine the effect of the SUMOylation-defective PB1/K612R mutation on the in vitro replication of IAV, we first compared the plaque morphologies of the wild-type WSN (H1N1), VN/1180 (H5N1), and AH/1 (H7N9) viruses with those of the SUMOylation-defective PB1/K612R mutant viruses in MDCK cells. Compared with the wild-type viruses, which formed large plaques, the PB1/K612R mutant viruses formed much smaller plaques, indicating that virus growth was greatly impaired by the lack of SUMOylation of PB1 at position 612 (Fig 7D–7F).

We next examined the replication kinetics of the PB1/K612R mutant viruses. MDCK cells were infected with one of the wild-type viruses or with the PB1/K612R mutant viruses at an MOI of 0.001. Supernatants were collected at 12, 24, 36, and 48 h p.i. and titrated by a plaque assay on MDCK cells. We found that the PB1/K612R mutation led to 25- to 76-, 2- to 7-, and 3- to 22-fold reductions in growth titers from 12 to 48 h p.i. for the WSN-PB1_K612R_ (H1N1), VN/1180-PB1_K612R_ (H5N1), and AH/1-PB1_K612R_ (H7N9) viruses, respectively, compared with the corresponding wild-type viruses. These data further demonstrate that the SUMOylation of PB1 at K612 is critical for optimal replication of IAV (Fig 7G–7I).

### The K612R mutation impairs the ability of PB1 to bind vRNA

**Fig 8:**
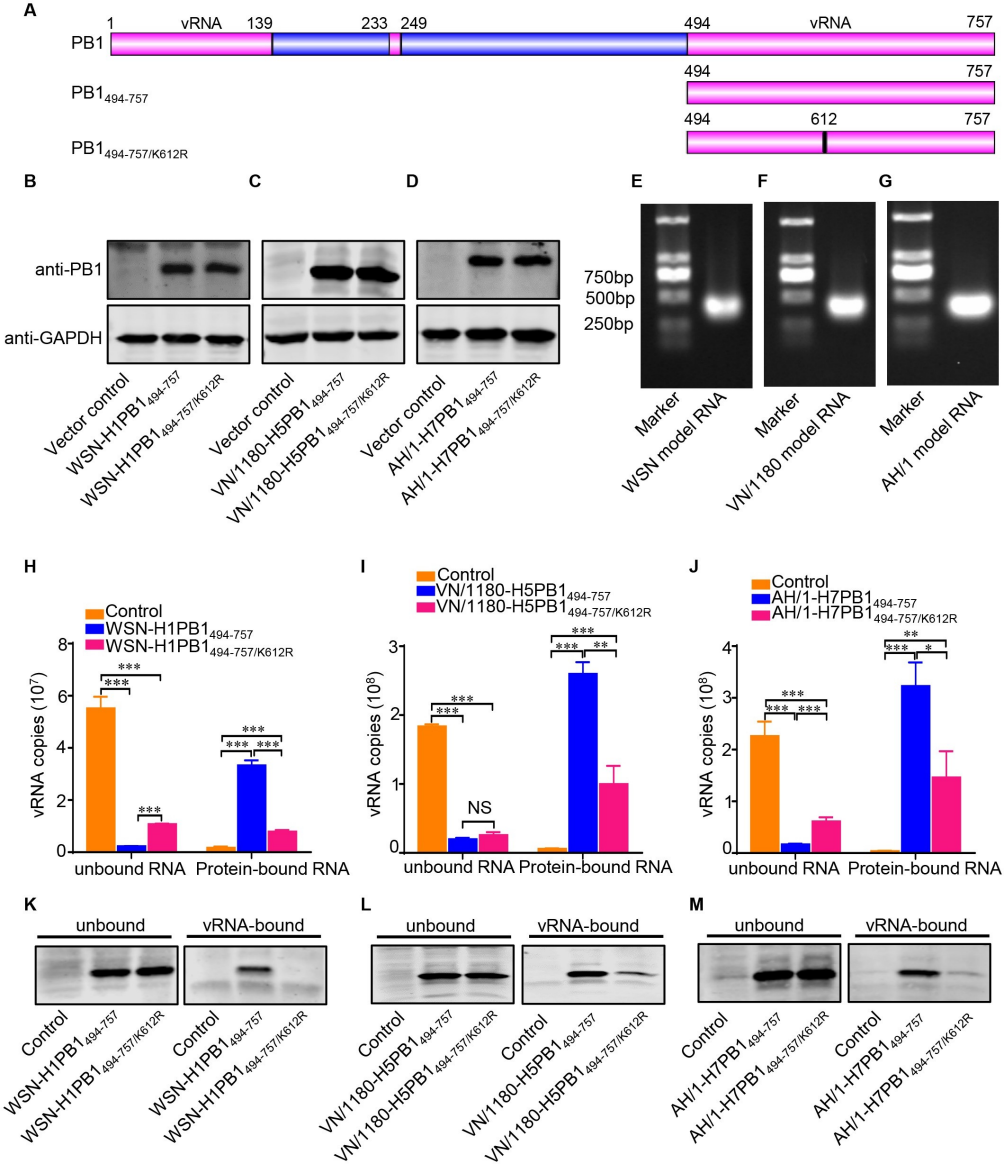
K612R mutation impairs the ability of PB1 to bind to vRNA. (A) Schematic illustration of IAV PB1 and its C-terminal 494–757 region that is involved in the vRNA-binding activity of PB1. The vRNA binding regions in PB1 protein are marked in pink. (B-D) HEK293T cells were transfected with plasmids expressing the Flag-tagged 494–757 region of wild-type PB1 or a K612R mutant PB1 of WSN (H1N1) (B), VN/1180 (H5N1) (C), or AH/1 (H7N9) (D) virus; plasmid expression was verified by western blotting. (E-G) Model vRNAs were transcribed from the NS gene of the WSN (H1N1) (E), VN/1180 (H5N1) (F), or AH/1 (H7N9) (G) virus in vitro, and detected by gel electrophoresis. (H-J) A protein binding vRNA assay was performed to quantify the amount of vRNA bound by the truncated wild-type or mutant PB1 proteins. The amount of vRNA bound by WSN-H1PB1_494-757_ or WSN-H1PB1_494-757/K612R_ (H), VN/1180-H5PB1_494-757_ or VN/1180-H5PB1_494-757/K612R_ (I), or AH/1-H7PB1_494-757_ or AH/1-H7PB1_494-757/K612R_ (J) was quantified by use of qRT-PCR. (K-M) An RNA-protein pull-down assay was performed to quantify the amount of truncated wild-type or mutant PB1 protein that was bound by the model vRNA. The amount of truncated PB1 protein WSN-H1PB1_494-757_ or WSN-H1PB1_494-757/K612R_ (K), VN/1180-H5PB1_494-757_ or VN/1180-H5PB1_494-757/K612R_ (L), or AH/1-H7PB1_494-757_ or AH/1-H7PB1_494-757/K612R_ (M) bound and unbound by the model vRNA was detected by western blotting. Data shown are representative of three independent experiments in (H-M). The results are expressed as the mean ± SD of triplicates and the significance was tested with a one-way ANOVA followed by t-test (H-J). *, *P* < 0.05; **, *P* < 0.01; ***, *P* < 0.001.

The C-proximal sequences located downstream of position 493 (494–757) of PB1 binds to vRNA [33]. The amino acid K612 is located in the vRNA binding domain (Fig 8A). We therefore determined whether the SUMOylation-defective K612R mutation affects the ability of PB1 to bind to vRNA. HEK293T cells were transfected with plasmid expressing the Flag-tagged 494–757 region of wild-type PB1 or the PB1/K612R mutant of WSN (H1N1), VN/1180 (H5N1), or AH/1 (H7N9) virus (Fig 8B–8D). A model vRNA was transcribed from the NS gene of the WSN (H1N1), VN/1180 (H5N1), or AH/1 (H7N9) virus in vitro (Fig 8E–8G), and the protein-vRNA binding assay was carried out as previously reported [34]. As shown in Fig 8H, the amount of vRNA bound by WSN-H1PB1_494-757_ protein was much higher than that bound by WSN-H1PB1_494-757/K612R_. When tested in the VN/1180 (H5N1) or AH/1 (H7N9) background, the VN/1180-H5PB1_494-757_ and AH/1-H7PB1_494-757_ proteins again bound much more model vRNA than the corresponding PB1/K612R mutant proteins, VN/1180-H5PB1_494-757/K612R_ and AH/1-H7PB1_494-757/K612R_ (Fig 8I and 8J). These data demonstrate that the K612R mutation impairs the vRNA-binding ability of PB1.

To further confirm that the K612R mutation affects the binding of the PB1 protein to vRNA, we conducted an RNA-protein pull-down experiment. Ten micrograms of biotinylated model vRNA was captured with streptavidin magnetic beads. Cell lysates containing 100 μg of total protein that had previously been transfected with a plasmid expressing truncated WSN-H1PB1 protein (PB1_494-757_ or PB1_494-757/K612R_) or pCAGGS vector as a control were then added to the RNA-bound beads. The amount of both unbound and vRNA-bound proteins was analyzed by western blotting. We found that the same amount of vRNA bound more WSN-H1PB1_494-757_ protein than WSN-H1PB1_494-757/K612R_ protein (Fig 8K). When we conducted this experiment in the VN/1180 (H5N1) and AH/1 (H7N9) backgrounds, we found that the amount of the PB1/K612R protein bound by vRNA was significantly lower than that of the wild-type protein (Fig 8L and 8M). Collectively, these results demonstrate that the K612R mutation in IAV PB1 impairs the ability of the PB1 protein to bind vRNA.

### The virulence of SUMOylation-defective PB1/K612R mutant viruses is attenuated in mice

**Fig 9:**
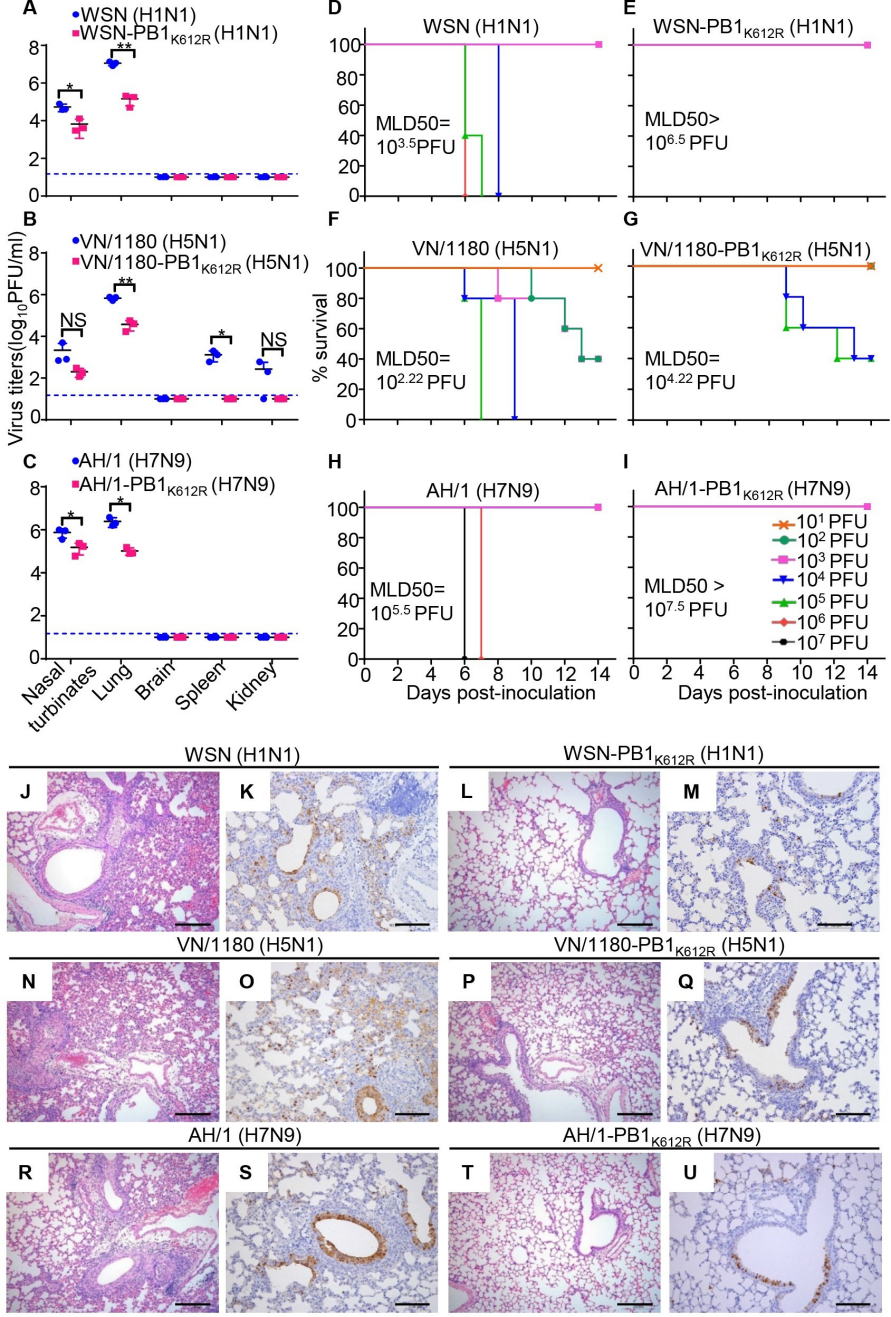
SUMOylation-defective PB1/K612R mutation attenuates the replication and pathogenicity of IAVs in mice. (A) Replication of WSN (H1N1) and WSN-PB1_K612R_ (H1N1) in mice. Six-week-old BALB/c mice were inoculated intranasally with 10^6^ PFU of WSN (H1N1) or WSN-PB1_K612R_ (H1N1), and organs were collected on day 3 p.i. for virus titration by plaque assays (n = 3). Data shown are mean ± SD for organ samples from three mice; significance was assessed with a one-way ANOVA followed by t-test. *, *P* < 0.05; **, *P* < 0.01. NS, not significant. (B) Replication of VN/1180 (H5N1) and VN/1180-PB1_K612R_ (H5N1) in mice inoculated with 10^5^ PFU of the indicated virus, as determined in (A). (C) Replication of AH/1 (H7N9) and AH/1-PB1_K612R_ (H7N9) in mice inoculated with 10^6^ PFU of the indicated virus, as determined in (A). (D, E) Mortality assessment of mice infected with WSN (H1N1) (D) or WSN-PB1_K612R_ (H1N1) (E). Mice were inoculated intranasally with different doses of the indicated viruses and observed for 14 days. (n = 5 mice for each group). (F, G) Mortality assessment of mice infected with VN/1180 (H5N1) (F) and VN/1180-PB1_K612R_ (H5N1) (G) as determined in (D, E). (H, I) Mortality assessment of mice infected with AH/1 (H7N9) (H) and AH/1-PB1_K612R_ (H7N9) (I) as determined in (D, E). (J-M) Hematoxylin-and-eosin (H&E) staining (J, L) and immunohistochemical (IHC) staining for viral NP (K, M) of lung sections prepared on day 3 p.i. from mice intranasally infected with 10^6^ PFU of WSN (H1N1) or WSN-PB1_K612R_ (H1N1). (N-Q) H&E staining (N, P) and IHC staining for viral NP (O, Q) of lung sections prepared on day 3 p.i. from mice intranasally infected with 10^5^ PFU of VN/1180 (H5N1) or VN/1180-PB1_K612R_ (H5N1). (R-U) H&E staining (R, T) and IHC staining for viral NP (S, U) of lung sections prepared on day 3 p.i. from mice intranasally infected with 10^6^ PFU of AH/1 (H7N9) or AH/1-PB1_K612R_ (H7N9). Scale bars = 200 μm (J, L, N, P, R, and T); Scale bars = 100 μm (K, M, O, Q, S, and U).

To determine the significance of SUMOylation at K612 of PB1 to the virulence of IAVs, we evaluated the replication of the WSN-PB1_K612R_ (H1N1), VN/1180-PB1_K612R_ (H5N1) and AH/1-PB1_K612R_ (H7N9) mutant viruses in mice. Three 6-week-old female BALB/c mice were inoculated with 10^6^ PFU of WSN (H1N1) or WSN-PB1_K612R_ (H1N1) mutant virus, or 10^5^ PFU of VN/1180 (H5N1) or VN/1180-PB1_K612R_ (H5N1) mutant virus, or 10^6^ PFU of AH/1 (H7N9) or AH/1-PB1_K612R_ (H7N9) mutant virus. The mice were killed on day 3 p.i., and the virus titers in their organs were determined by using plaque assays on MDCK cells. The WSN (H1N1) and WSN-PB1_K612R_ (H1N1) mutant viruses replicated only in respiratory organs, and the titers of the WSN (H1N1) virus in the nasal turbinates and lungs of the mice were significantly higher than those in these tissues of the mice that were inoculated with the WSN-PB1_K612R_ (H1N1) mutant virus (Fig 9A). The VN/1180 (H5N1) virus caused systemic infection, with virus replication detected in the nasal turbinates, lungs, spleens, and kidneys, whereas the replication of the VN/1180-PB1_K612R_ (H5N1) mutant virus was restricted to the respiratory tract, with much lower titers in the nasal turbinates and lungs than the wild-type VN/1180 (H5N1) virus (Fig 9B). Similarly, the viral titers of AH/1-PB1_K612R_ (H7N9) mutant virus in the nasal turbinates and lungs of mice were much lower than those inoculated with AH/1 (H7N9) virus (Fig 9C). These results demonstrate that the SUMOylation-defective PB1/K612R mutation significantly attenuates the replication of IAVs in vivo.

We next determined the 50% mouse lethal dose (MLD_50_) of the PB1/K612R mutant viruses. Groups of five mice were intranasally inoculated with serially diluted mutant viruses or the corresponding wild-type viruses, and their body weight, disease signs, and death were monitored daily for 14 days. The wild-type WSN (H1N1) virus caused severe disease and killed the mice at an MLD_50_ of 10^3.5^ PFU (Figs 9D and S2A). However, no deaths were observed among the mice inoculated with any dose of the WSN-PB1_K612R_ (H1N1) mutant virus, and all of the mice had gained weight at the end of the observation period (S2B Fig), yielding an MLD_50_ of >10^6.5^ PFU (Fig 9E). Consistent with these results, mice inoculated with the VN/1180-PB1_K612R_ (H5N1) mutant virus lost much less body weight than those inoculated with the same dose of the wild-type VN/1180 virus (S2C and S2D Fig); the MLD_50_ of the VN/1180 (H5N1) virus was 10^2.22^ PFU, whereas the lethality of the VN/1180-PB1_K612R_ (H5N1) mutant virus was reduced by 100-fold (MLD_50_ = 10^4.22^ PFU) (Fig 9F and 9G). Similarly, the body weight loss of mice inoculated with AH/1 (H7N9) virus and AH/1-PB1_K612R_ (H7N9) mutant virus were observed in the 10^5^–10^7^ PFU groups, with more severe body weight loss observed in mice inoculated with AH/1 (H7N9) virus than AH/1-PB1_K612R_ (H7N9) mutant virus (S2E and S2F Fig); and the MLD_50_ was 10^5.5^ PFU for AH/1 (H7N9) virus, whereas was >10^7.5^ PFU for AH/1-PB1_K612R_ (H7N9) mutant virus, displaying an at least 100-fold reduction compared with the wild-type virus (Fig 9H and 9I). These data indicate that the virulence of the SUMOylation-defective PB1/K612R mutant viruses is highly compromised compared with that of the wild-type viruses in mice.

We also performed histopathological studies on lung sections from the wild-type- and PB1/K612R mutant virus-inoculated mice. The WSN (H1N1) virus-infected mice exhibited more severe lung lesions (i.e. perivascular edema, infiltration of inflammatory cells, necrosis, and exfoliation of bronchiole epithelial cells) compared with the mice infected with the WSN-PB1_K612R_ (H1N1) mutant virus. Immunohistochemical analysis with an anti-NP pAb revealed more viral antigen-positive cells in the lungs of mice infected with the wild-type WSN (H1N1) virus than in mice infected with the mutant virus (Fig 9J–9M). Similar results were observed in the lungs of mice infected with the wild-type VN/1180 (H5N1), AH/1 (H7N9) and their corresponding PB1/K612R mutant viruses (Fig 9N–9U). These data show that the PB1/K612R mutant viruses induce significantly less severe lung lesions compared with the wild-type viruses. Collectively, the above findings indicate that the SUMOylation at PB1 K612 plays a pivotal role in the pathogenesis of IAVs in mice.

### SUMOylation at PB1 K612 may facilitate the replication and respiratory droplet transmission of IAV in ferrets

**Fig 10:**
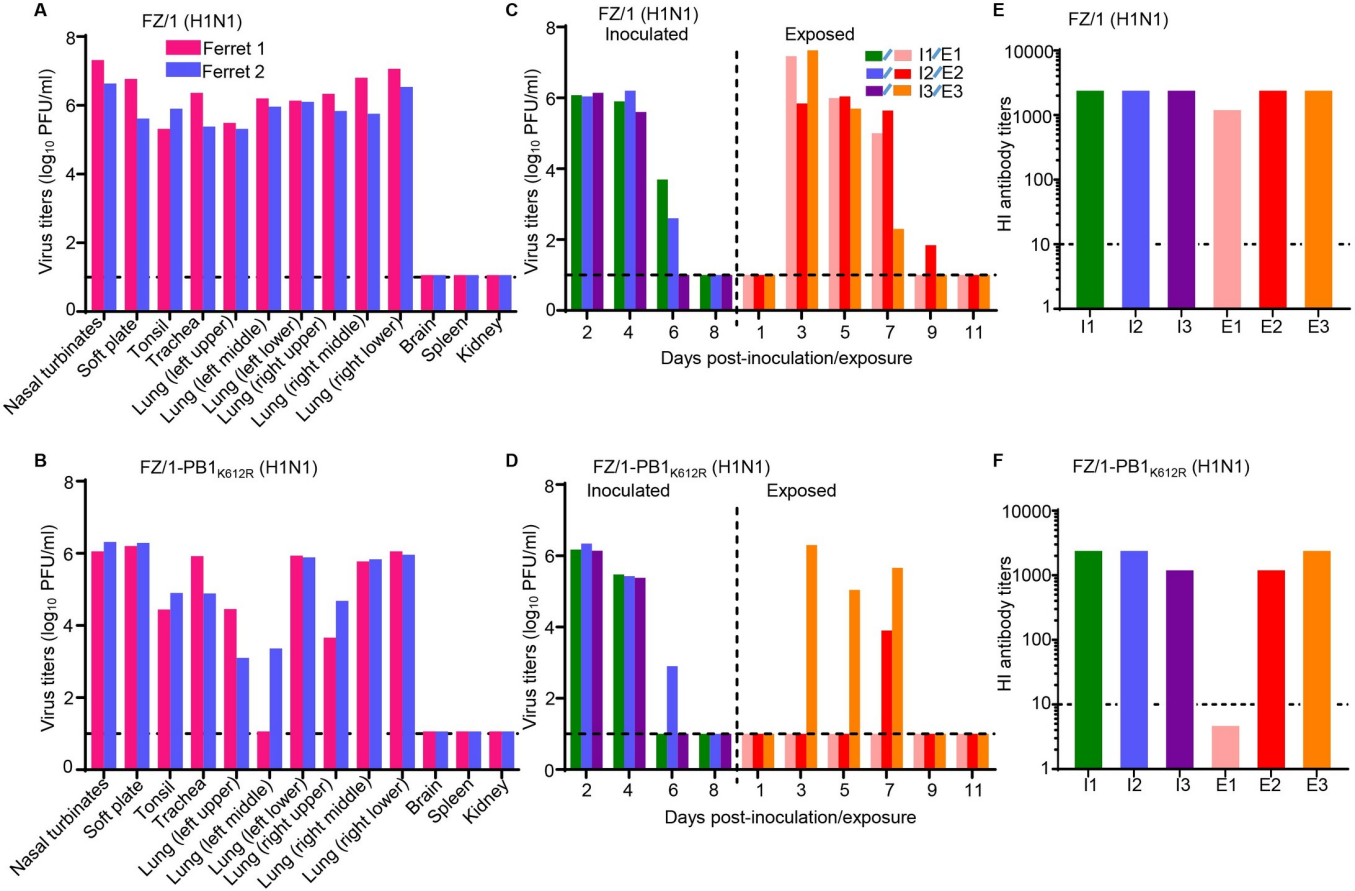
PB1/K612R mutation compromises the replication and respiratory droplet transmission of IAVs in ferrets. (A, B) Replication of a 2009 pandemic H1N1 virus FZ/1 and its mutant FZ/1-PB1_K612R_ in ferrets. Groups of two ferrets were intranasally inoculated with 10^6^ PFU of FZ/1 (A) or FZ/1-PB1_K612R_ (B). The ferrets were euthanized on day 4 post-inoculation and the indicated organs were collected for virus titration by plaque assays. Each color bar represents the virus titer from an individual animal. (C, D) Respiratory droplet transmission of FZ/1 (H1N1) (C) and FZ/1-PB1_K612R_ (H1N1) (D) in ferrets. Groups of three ferrets were intranasally inoculated with 10^6^ PFU of the tested virus, and 24 h later three naïve ferrets were introduced into the neighboring cages. Nasal washes were collected every 2 days for virus titration by plaque assays. Each color bar represents the virus titer from an individual ferret. (E, F) Seroconversion was confirmed by use of a hemagglutinin inhibition (HI) assay. Each bar represents the HI antibody titer from an individual animal. The dashed black lines indicate the lower limit of detection.

Efficient and sustained transmission among humans is the most prominent property of pandemic and epidemic IAVs [35]. To determine whether the SUMOylation of PB1 at K612 affects the transmissibility of IAVs, a 2009 pandemic H1N1 virus, A/Fuzhou/1/2009 (FZ/1, H1N1), and its SUMOylation-defective PB1/K612R mutant FZ/1-PB1_K612R_ (H1N1) were rescued by reverse genetics. We then tested the replication ability of these viruses in ferrets. Both the FZ/1 (H1N1) and FZ/1-PB1_K612R_ (H1N1) viruses were detected in the nasal turbinates, soft palate, tonsils, trachea, and lungs of the inoculated ferrets on day 4 p.i. Overall, the titers of the FZ/1-PB1_K612R_ (H1N1) virus in the organs tested were lower than those of the FZ/1 (H1N1) virus, especially in nasal turbinates, tonsils, and lungs (Fig 10A and 10B). We next tested the respiratory droplet transmissibility of the FZ/1 (H1N1) and FZ/1-PB1_K612R_ (H1N1) viruses in ferrets. Both the FZ/1 (H1N1) and FZ/1-PB1_K612R_ (H1N1) viruses were detected in nasal washes from inoculated ferrets between days 2 and 6 p.i. Notably, FZ/1 (H1N1) was present in the nasal washes of all three exposed ferrets on days 3, 5, and 7 post-exposure (p.e.), and was present in the nasal wash of one exposed ferret on day 9 p.e., with peak titers ranging from 6.0 to 7.3 log_10_ PFU/mL (Fig 10C). By contrast, in the FZ/1-PB1_K612R_ (H1N1)-exposed group, the virus was detected in the nasal wash of only one ferret on day 3 p.e., and in the nasal wash of another ferret until day 7 p.e., with peak titers ranging from 3.9 to 6.3 log_10_ PFU/mL (Fig 10D). All of the inoculated ferrets and the exposed ferrets that shed viruses seroconverted on day 21 p.i. or p.e. (Fig 10E and 10F). These results suggest that the SUMOylation-defective PB1/K612R mutation is correlated with the impaired replication and transmission of the FZ/1 (H1N1) virus in ferrets.

Finally, we sought to determine whether the introduced PB1/K612R mutation was stably maintained during viral replication in ferrets. Viral RNAs extracted from nasal washes of FZ/1-PB1_K612R_-inoculated and -exposed ferrets were reverse-transcribed into cDNA, and the full-length PB1 gene was amplified and deep-sequenced. Strikingly, PB1/K612R was not stably maintained during virus replication in ferrets. The R612K reversion mutation in PB1 was found on day 2 p.i. in the inoculated ferrets, and all of the viruses contained the R612K reversion mutation in the inoculated animals on day 4 p.i. (S3 Table). In the virus-positive exposed ferret on day 3 p.e., 99% of the viruses in the nasal wash contained PB1 R612. However, the viruses mutated to K612 quickly, with 96% of the sequencing reads bearing K612 on day 5 p.e. Interestingly, the second virus-positive exposed ferret had already acquired the PB1 R612K reversion mutation when the virus was detected in the nasal wash on day 7 p.e. (Fig 10D and S3 Table). These results suggest that the transmission of the FZ/1-PB1_K612R_ (H1N1) virus may have occurred or have been enhanced after it acquired the R612K reversion mutation in its PB1 protein.

## Discussion

Posttranslational modifications, such as phosphorylation, acetylation, ubiquitination, and SUMOylation, play important roles in the regulation of protein activity, localization and stability [36]. SUMOylation is an important reversible posttranslational modification that is extensively involved in the regulation of viral protein functions [37]. Several viral proteins of IAV, for example, NS1, M1, and NP, are reported to be SUMOylated in transfected and infected cells [23,25–27,38]. As the assembly core of the IAV polymerase complex, PB1 binds to the PA protein through its N-terminal domain and binds to the PB2 protein through its C-terminal domain in order to play a central role in the transcription and replication of the viral genome. In addition to the clear role of SUMOylation in the posttranslational modification of the specific IAV proteins mentioned above, IAV infection has been shown to induce a global increase in cellular SUMOylation levels and PB1 has been suggested to be a target of SUMOylation [27,28]. However, the molecular details of PB1 SUMOylation and its role in virus replication remain unclear. More importantly, the biological significance of SUMOylation for the pathogenesis and transmission of IAVs has never been investigated.

In the present study, we clearly demonstrated that IAV PB1 is SUMOylated by SUMO1 in transfected and infected cells, and we mapped the key SUMOylation site of the PB1 protein to residue K612 in the C-terminal region. Large-scale sequence analysis revealed that the lysine residue at position 612 of PB1 is highly conserved among different IAV strains. These findings reveal that SUMOylation at K612 of PB1 is a common posttranslational modification mechanism of IAVs. We speculate that the acquisition and stable maintenance of K612 in PB1 is the outcome of host adaptation of IAVs to achieve optimal viral fitness.

The functional consequences of SUMOylation are highly diverse depending on the target proteins [21,39,40]. We investigated the effect of SUMOylation of K612 on the biological properties of the PB1 protein, and found that SUMOylation at K612 did not affect the interaction of PB1 with the other two polymerase subunits, PB2 and PA, and also did not interfere with vRNP complex assembly. Unlike the viral NS1 protein, which is stabilized by SUMOylation [24], the SUMOylation of K612 had no effect on the stability of PB1, and also did not change the cellular localization of PB1 in infected cells. Functionally, we found that the vRNP complex activity decreased significantly when the PB1 protein could not undergo SUMO modification at the mutated K612R residue. In the WSN (H1N1) and VN/1180 (H5N1) virus backgrounds, the vRNP complex activity was reduced by about 50% due to the PB1/K612R mutation, and in the AH/1 (H7N9) virus background, this mutation led to an approximately 95% reduction in vRNP complex activity. Because the SUMO acceptor site K612 is located in the vRNA binding domain of the PB1 protein, we tested whether the K612R mutation affects the binding of PB1 to vRNA. We found that lack of SUMOylation of PB1 at the mutated K612R residue significantly impaired the ability of the PB1 protein to bind to vRNA, thereby severely limiting the replication and transcription of IAVs. These data clearly indicate that SUMOylation at K612 is indispensable to PB1 for the ability to bind to vRNA with high affinity, which is essential for PB1 to fulfil its function as the enzymatic core of the vRNP complex.

In characterizing the effect of SUMOylation at K612 of PB1 on the replication of IAVs, we found that SUMOylation-defective PB1 K612R mutant viruses formed much smaller plaques in MDCK cells than the wild-type viruses, and that their growth kinetics were also severely compromised in MDCK cells. These results are consistent with the role of SUMOylation of other IAV proteins, such as NP, M1, and NS1, for viral replication in vitro [24–26]. Consistent with the in vitro growth properties, the PB1/K612R mutant viruses replicated to much lower titers in mouse organs compared with the corresponding wild-type viruses; of note, the replication of the VN/1180-PB1_K612R_ (H5N1) mutant virus was restricted to respiratory organs, whereas the wild-type VN/1180 (H5N1) virus caused systemic infection.

The biological significance of SUMOylation at K612 of PB1 on the pathogenesis and transmission of IAVs was thoroughly explored. In the mouse model, we found that the virulence of the SUMOylation-defective PB1 K612R mutant viruses was significantly attenuated compared with that of the wild-type WSN (H1N1), VN/1180 (H5N1) or AH/1 (H7N9) virus, indicating that SUMOylation at K612 of PB1 is necessary for IAVs to be virulent in mice. Sustained respiratory droplet transmission is an essential property of pandemic and epidemic IAVs [35], and we found that the SUMOylation of PB1 at K612 may facilitate the transmission of IAVs. The wild-type 2009 pandemic H1N1 virus, A/Fuzhou/1/2009, transmitted to all three exposed ferrets via respiratory droplets, whereas the PB1-K612R mutant virus transmitted to two of the three exposed ferrets. Notably, the R612K reversion mutation was identified on day 2 p.i., and accounted for 100% of the residues at position 612 of PB1 on day 4 p.i., in all three inoculated ferrets; virus shedding in one of the two positive exposed ferrets was detected until day 7 p.e., and almost all shed viruses carried the R612K reversion mutation. These findings indicate that the stress condition, caused by the SUMOylation-defective PB1/K612R mutation, drives the reversion of the mutant virus to the wild-type phenotype. The SUMOylation at K612 of PB1 would, therefore, appear to be a prerequisite for the efficient replication and transmission of pandemic influenza viruses. Together, these data shed light on the importance of SUMOylation at K612 of PB1 in the pathogenesis and the transmission of IAVs.

In summary, here we found that IAVs can manipulate the host SUMOylation system to modify their PB1 protein. The highly conserved K612 residue of PB1 was pinpointed as the key acceptor site that conjugates with SUMO1. vRNP complex activity and viral replication were severely attenuated in the presence of the SUMOylation-defective PB1/K612R mutation, which were attributed to the impaired binding affinity to vRNA caused by this mutation. Strikingly, the SUMOylation at K612 of PB1 was important for the pathogenesis and transmission of IAVs in mammalian models. Collectively, our study revealed the biological significance of SUMOylation at K612 of PB1 in IAV infection, which could serve as a potential therapeutic target for the development of novel antiviral therapies.

## Materials and methods

### Ethics statements

This study was carried out in strict accordance with the recommendations in the Guide for the Care and Use of Laboratory Animals of the Ministry of Science and Technology of the People’s Republic of China. The protocols for animal studies were approved by the Committee on the Ethics of Animal Experiments of the Harbin Veterinary Research Institute (HVRI) of the Chinese Academy of Agricultural Sciences (CAAS) (approval numbers BRDW-XBS–19 for mice and BRDW-XD–19 for ferrets).

### Facility

All experiments with live H5N1 and H7N9 viruses were conducted within the enhanced animal biosafety level 3 (ABSL3+) facility at the HVRI of CAAS, which is approved for such use by the Ministry of Agriculture and Rural Affairs of the People’s Republic of China. All animal studies were approved by the Review Board of the HVRI, CAAS. The details of the facility and the biosafety and biosecurity measures used have been previously reported [31,41].

### Cells and viruses

HEK293T cells were cultured in DMEM (Sigma-Aldrich) supplemented with 10% fetal bovine serum (Sigma-Aldrich), human lung carcinoma cells (A549) were cultured in F12K medium (Life Technologies) supplemented with 10% FBS, and Madin-Darby canine kidney (MDCK) cells were cultured in MEM (Life Technologies) containing 5% newborn calf serum (Sigma-Aldrich). All cells were maintained in a humidified incubator containing 5% CO_2_ at 37°C.

A/chicken/Vietnam-Ca Mau/1180/2006 (VN/1180, H5N1) and A/Anhui/1/2013 (AH/1, H7N9) were grown in 10-day-old specific-pathogen-free (SPF) embryonated chicken eggs, as reported previously [6,42]. A/WSN/33 (WSN, H1N1) and a 2009 pandemic H1N1 virus, A/Fuzhou/1/2009 (FZ/1, H1N1), were propagated in MDCK cells cultured in MEM containing 0.3% bovine serum albumin (BSA, Sigma-Aldrich) and 0.5 μg/ml N-tosyl-L-phenylalanyl chloromethyl ketone (TPCK)-treated trypsin (Sigma-Aldrich).

### Plasmids

The PB2, PB1, PA, and NP genes of three influenza viruses [i.e. WSN (H1N1), VN/1180 (H5N1), and AH/1 (H7N9)] were cloned into the mammalian expression vector pCAGGS [43]. pCAGGS-Flag-WSN-H1PB1, pCAGGS-Flag-VN/1180-H5PB1, and pCAGGS-Flag-AH/1-H7PB1 were generated by inserting the PB1 open reading frames (ORFs) of the WSN (H1N1), VN/1180 (H5N1), and AH/1 (H7N9) viruses with a Flag tag sequence fused at the N-terminus into the pCAGGS vector. Truncation constructs of the three different PB1 proteins, WSN-H1PB1_494-757_, VN/1180-H5PB1_494-757_, AH/1-H7PB1_494-757_, as well as the corresponding K612R mutants, were generated by using a PCR approach. The full-length ORFs of human SUMO1, SUMO2, and SUMO3 were cloned into the pCAGGS vector with an HA tag at the N-terminus. SUMO1 was further subcloned into pCAGGS with a Flag tag at the N-terminus. SUMO1_mut_ was obtained by a PCR approach using primers containing GG-to-AA mutations at the C-terminus of SUMO1 as previously reported [26]. The ORFs of SENP1 and Ubc9 were amplified from human A549 cDNA and cloned into pCDNA3.1 and pCAGGS, respectively. The SENP1 catalytic mutant plasmid, pCDNA3.1-SENP1_mut_-V5 (SENP1_mut_), was generated from SENP1 by site-directed mutagenesis as described previously [44]. Plasmids expressing the WSN-H1PB1 mutants, including WSN-H1PB1/K379R, WSN-H1PB1/K612R, and WSN-H1PB1/K736R, were derived from pCAGGS-Flag-WSN-H1PB1 by PCR-based site-directed mutagenesis. By using a PCR approach, a K612R mutation was introduced into different viral PB1 genes, which were then cloned into the pHH21 vector [15]. The plasmid pHH21-SC09NS F-Luc, for the expression of a viral RNA-like firefly luciferase gene under the control of the human RNA polymerase I promoter, has been reported previously [30,31]. All constructs were sequenced to ensure the absence of unwanted mutations. The primer sequences used for cloning are available upon request.

### Antibodies

Rabbit anti-NP polyclonal antibody (pAb) and mouse monoclonal antibodies (mAbs) against IAV PB1, PB2, PA, and NP were prepared and stored in our laboratory [30,45]. The following primary antibodies were purchased from commercial sources: rabbit anti-SUMO-1 (C9H1) mAb (4940, Cell Signaling Technology), rabbit anti-PB1 pAb (GTX125923, GeneTex), anti-Flag M2 affinity gel (A2220, Sigma-Aldrich), mouse anti-GAPDH mAb (60004-1-Ig, Proteintech), and rabbit anti-GAPDH pAb (10494-1-AP, Proteintech). The secondary antibodies DyLight 800 goat anti-mouse IgG (H+L) (072-07-18-06) and DyLight 800 goat anti-rabbit IgG (H+L) (072-07-15-06), purchased from KPL (Gaithersburg, MD), were used for western blotting. The secondary antibody Alexa Fluor 488 donkey anti-rabbit IgG (H+L) (A21206, Thermo Fisher Scientific) was used in the immunofluorescence assay, and horseradish peroxidase-conjugated goat anti-rabbit IgG (A9169, Sigma-Aldrich) was used in the immunohistochemical assay.

### Generation of mutant viruses by reverse genetics

Mutant viruses with the PB1/K612R mutation (AAA at positions 1858–1860 of PB1 gene were mutated to CGA) in the WSN (H1N1), VN/1180 (H5N1), AH/1 (H7N9) and FZ/1(H1N1) background were generated by using the reverse genetics system as described previously [15]. The rescued viruses were fully sequenced to ensure the absence of unwanted mutations.

### Co-IP and western blotting analysis

HEK293T cells were transfected for 48 h with the indicated plasmids by using Lipofectamine 2000 reagent (Invitrogen). To determine the SUMOylation of the PB1 protein during viral infection, HEK293T cells were first transfected for 24 h with Ubc9, SUMO1, or SUMO1_mut_, followed by infection with WSN (H1N1) or WSN-PB1_K612R_ (H1N1), VN/1180 (H5N1) or VN/1180-PB1_K612R_ (H5N1), and AH/1 (H7N9) or AH/1-PB1_K612R_ (H7N9) mutant virus (MOI = 2) or mock infection for 18 h. The transfected or infected cells were then washed with cold PBS and lysed with NP40 buffer (Beyotime Biotechnology, China) containing complete protease inhibitor cocktails (Roche Diagnostics) for 30 min on ice. The cell lysates were centrifuged at 12,000 rpm at 4°C for 10 min, and the supernatant was then subjected to immunoprecipitation with anti-Flag agarose at 4°C for 6 h. The beads were washed four times with cold PBS (containing 1 mM PMSF), and the bound proteins were eluted with SDS loading buffer and separated by SDS-PAGE. The proteins were transferred onto nitrocellulose membranes (GE Healthcare), which were blocked in 5% skimmed milk in PBST for 1 h, and then incubated with the indicated primary and secondary antibodies. Blots were visualized with the Odyssey infrared imaging system (Li-Cor BioScience).

### Immunofluorescence assay

A549 cells seeded in glass-bottom dishes were infected with WSN (H1N1) or WSN-PB1_K612R_ (H1N1) mutant virus at an MOI of 5. At 2, 4, 6, 8, and 10 h p.i., the cells were fixed with 4% paraformaldehyde for 1 h and permeabilized with 0.1% Triton X-100 in PBS for 15 min. The permeabilized cells were blocked with 5% bovine serum albumin in PBS for 1 h, and then were incubated with rabbit anti-PB1 pAb for 1 h. After three washes with PBS, the cells were incubated with the secondary antibody Alexa Fluor 488 donkey anti-rabbit IgG (H+L) for 1 h, followed by incubation with 4’,6-diamidino-2-phenylindole (DAPI, Sigma-Aldrich) for 15 min to stain the nuclei. Images were acquired by using a Zeiss LSM880 confocal microscope.

### Plaque assay

Plaque assays were performed on MDCK cells in 12-well plates as described previously [43]. Briefly, MDCK cells were infected with 10-fold serial dilutions of virus supernatants in 1×MEM (0.3% BSA) for 1 h at 37°C. Then the cells were washed with PBS and overlaid with 1% SeaPlaque agarose (Lonza) in 1×MEM (0.3% BSA, 0.5 μg/ml TPCK-treated trypsin). After 48 to 72 h of incubation, the cells were fixed with formalin, and plaques were stained with 0.1% crystal violet and counted.

### Dual-luciferase reporter assay

HEK293T cells were transfected with pCAGGS constructs expressing viral PB2, PB1 (wild-type or K612R mutant), PA, and NP proteins (0.5 μg each), the construct pHH21-SC09NS F-Luc (0.1 μg), and an internal control pRL-TK (0.05 μg, Promega) by using Lipofectamine LTX & Plus reagents (Invitrogen). Cells were incubated at 37°C for 48 h, and cell lysates were subsequently prepared by using the Dual-Luciferase Reporter Assay System (Promega). The luciferase activities were measured on a GloMax 96 microplate luminometer (Promega) as reported previously [30–32].

### Generation of model vRNA

A 360-nucleotide model vRNA was transcribed *in vitro* from a cDNA containing the T7 promoter and the noncoding region of the NS gene of WSN (H1N1), VN/1180 (H5N1), or AH/1 (H7N9) virus by using the RiboMax Large Scale RNA Production Systems (Promega) as described previously [34]. The vRNAs transcribed in vitro were concentrated and purified by using a Spin Column RNA Cleanup & Concentration Kit (Sangon Biotech) and quantified by using NanoDrop (ThermoFisher).

### Protein binding vRNA assay

A protein binding vRNA assay was carried out as described previously [34]. Briefly, HEK293T cells were transfected with a plasmid expressing Flag-tagged truncated PB1_494-757_ or PB1_494-757/K612R_ or with pCAGGS-Flag vector as a control. The cells were lysed in RIPA lysis buffer (ThermoFisher) supplemented with protease inhibitor cocktail (Roche). Anti-Flag agarose was added to cell lysates containing 100 μg of total protein, followed by incubation for 4 h on a roller at 4°C. After three washes with diethyl pyrocarbonate (DEPC)-treated 0.1M NaCl, vRNA (3 μg) transcribed in vitro was added to the beads and incubated on a roller for 4 h at 4°C. The supernatant containing the unbound vRNA and the beads bearing the protein-bound vRNA were isolated and quantified by means of real-time RT-PCR (primer sequences available upon request).

### RNA-protein pull-down assay

The RNA binding protein assay was performed according to the instructions of the Pierce Magnetic RNA-Protein Pull-Down Kit (20164, ThermoFisher). In brief, 10 μg of model vRNA was labeled with a single desthiobiotinylated cytidine bisphosphate at the 3’ end by using T4 RNA ligase. Then, the labeled vRNA was captured with streptavidin magnetic beads. After the beads were washed twice with 20 mM Tris (pH7.5) and Protein-RNA binding buffer, cell lysates containing 100 μg of total protein were added to the RNA-bound beads and incubated for 1 h at 4°C with rotation. The beads containing the biotinylated RNA/protein complexes were collected on a magnetic stand. The supernatants were harvested for subsequent detection and analysis. The beads were then washed three times with washing buffer and boiled in SDS loading buffer. Both the supernatants and the precipitates were resolved by means of 10% SDS-PAGE followed by western blotting.

### Mouse study

To evaluate the replication and pathogenicity of viruses in mice, groups of three 6-week-old female BALB/c mice (Vital River Laboratories, China) were inoculated intranasally with 10^6^ PFU of WSN (H1N1) or WSN-PB1_K612R_ (H1N1) mutant virus, 10^5^ PFU of VN/1180 (H5N1) or VN/1180-PB1_K612R_ (H5N1) mutant virus, or 10^6^ PFU of AH/1 (H7N9) or AH/1-PB1_K612R_ (H7N9) mutant virus. The mice were euthanized on day 3 p.i., and their organs, including nasal turbinates, lungs, spleens, kidneys and brains, were collected and titrated for virus infectivity by use of plaque assays on MDCK cells. Simultaneously, a lobe of lung tissue was fixed in 10% neutral buffered formalin and histopathologically examined.

To determine the 50% mouse lethal dose (MLD_50_) of the wild-type and mutant viruses, groups of five 6-week-old female BALB/c mice were lightly anesthetized with CO_2_ and inoculated intranasally with 10-fold serial dilutions containing 10^3^−10^6^ PFU of WSN (H1N1) or WSN-PB1_K612R_ (H1N1) mutant virus, 10^1^−10^5^ PFU of VN/1180 (H5N1) or VN/1180-PB1_K612R_ (H5N1) mutant virus, or 10^3^−10^7^ PFU of AH/1 (H7N9) or AH/1-PB1_K612R_ (H7N9) mutant virus, in a volume of 50 μl. The mice were monitored daily for 14 days for weight loss and mortality. The MLD_50_ was calculated by using the method of Reed and Muench.

### Histopathology

Lung samples from mice infected with WSN (H1N1) or WSN-PB1_K612R_ (H1N1), VN/1180 (H5N1) or VN/1180-PB1_K612R_ (H5N1), and AH/1 (H7N9) or AH/1-PB1_K612R_ (H7N9) were collected on day 3 p.i., fixed in 10% neutral buffered formalin, embedded in paraffin, and cut into 4-μm sections. The sections were stained with hematoxylin-eosin (H&E) or used in immunohistochemical (IHC) assays with a rabbit anti-NP pAb and horseradish peroxidase-conjugated goat anti-rabbit IgG.

### Ferret study

Four-month-old female ferrets (Wuxi Cay Ferret Farm, China) that were serologically negative for influenza viruses were used in this study. The animals were anesthetized via intramuscular injection with ketamine (20 mg/kg) and xylazine (1 mg/kg). To investigate virus replication, groups of two ferrets were anesthetized and inoculated i.n. with 10^6^ PFU of test virus in a 500-μl volume (250 μl per nostril). The ferrets were euthanized on day 4 p.i. and their nasal turbinates, soft palate, tonsils, trachea, lung, spleen, kidneys, and brain were collected for virus titration. For the respiratory droplet transmission studies, groups of three ferrets were inoculated i.n. with 10^6^ PFU of test virus and housed in specially designed cages inside an isolator as described previously [6,7,46]. Twenty-four hours later, three naïve ferrets were placed in an adjacent cage (4 cm away), separated by a double-layered net divider. Nasal washes were collected from each of the virus-infected ferrets on days 2, 4, 6, and 8 p.i. and from each of the exposed ferrets on days 1, 3, 5, 7, 9, and 11 p.e., and were titrated by use of plaque assays on MDCK cells. Sera were collected from all animals on day 21 p.i. or p.e. for HI antibody detection.

### Deep sequencing

Viral RNA was extracted from the stock virus, nasal washes, or lung homogenates by using the QIAmp viral RNA mini kit (QIAGEN) and was reverse-transcribed into cDNA by use of Uni12 primer (5’-AGCRAAAGCAGG-3’). The genome of the viruses was amplified and sequenced as described previously [31]. Briefly, next generation sequencing libraries were constructed by using the NEBNext Ultra DNA Library Prep Kit for Illumina. For each sample, 1 μg of DNA was randomly fragmented to <500 bp by sonication. The fragments were then treated with End Repair Mix for end repairing and with A-Tailing Mix for dA-tailing, followed by a T-A ligation to add adaptors to both ends. Each sample was then amplified by PCR using P5 and P7 primers. The PCR products were cleaned and quantified by using a Qubit2.0 Fluorometer (Invitrogen). Then, libraries with different indices were multiplexed and loaded onto an Illumina HiSeq instrument. Sequencing was carried out using a 2×150 paired-end configuration; image analysis and base calling were conducted by the HiSeq Control Software (HCS) + OLB + GAPipeline-1.6 (Illumina) on the HiSeq instrument. The genomes of all of the samples sequenced yielded more than 5000-fold genome coverage depth (with raw sequencing data of approximately 2.0 Gb obtained per sample). The sequences were processed and analyzed by GENEWIZ (GENEWIZ Biotechnology).

### Statistical analysis

Quantitative data are presented as means ± SD of at least three biological replicates. Data were statistically analyzed with a two-tailed unpaired Student’s t-test or a one-way ANOVA followed by t-test by using GraphPad Prism 7.0 software. Statistical parameters are reported in the figures and figure legends. *P* values < 0.05 were considered statistically significant.

## Supporting information

S1 Table Analysis of the SUMO acceptor lysine residue at position 612 (K612) of PB1 protein in different subtypes of influenza A virus. (DOCX)

S2 Table Frequency of the indicated amino acid at position 612 of PB1 protein of the indicated viruses. (DOCX)

S3 Table Assessment of the amino acid phenotypes at position 612 of PB1 protein in the viruses recovered from the nasal washes of FZ/1-PB1_K612R_ (H1N1) inoculated or exposed ferrets. (DOCX)

S1 Fig SUMOylation at K612 of PB1 has no effect on the assembly of the vRNP complex of IAV. (A) HEK293T cells were cotransfected with plasmids encoding PB2, PB1 or PB1/K612R, PA, and NP of WSN (H1N1) virus, together with the pHH21-SC09NS F-Luc construct. Forty-eight hours later, cell lysates were immunoprecipitated with a mouse anti-PB1 mAb, followed by western blotting to detect the vRNP components, PB2, PA, and NP. (B) Quantification of immunoprecipitated PB2, PA, NP in (A) by using ImageJ software. The amounts of precipitated PB2, PA, and NP were normalized to the amount of precipitated PB1. The results are expressed as the mean ± SD of three assays and the significance was tested with a multiple t test (B). NS, not significant.
(TIF)

S2 Fig Body weight change of mice inoculated with wild-type or PB1_K612R_ mutant viruses. (A-F) Five mice per group were inoculated intranasally with the indicated doses of WSN (H1N1) (A), WSN-PB1_K612R_ (H1N1) (B), VN/1180 (H5N1) (C), VN/1180-PB1_K612R_ (H5N1) (D), AH/1 (H7N9) (E), or AH/1-PB1_K612R_ (H7N9) (F) virus. Body weights were monitored daily for 14 days.
(TIF)
