# Supplementary material for: Viral RNA-binding ability conferred by SUMOylation at PB1 K612 of influenza A virus is essential for viral pathogenesis and transmission
Source: PLoS Pathog. 2021 Feb 11;17(2):e1009336. doi: 10.1371/journal.ppat.1009336 (PMC7904188; doi:10.1371/journal.ppat.1009336)
Supplement: S1 Table — (DOCX) [file ppat.1009336.s001.docx]

| Influenza A Virus of different subtypes |  | No. of isolates analyzed^a^ |  | Percentage of PB1 proteins with K612 residue (%) |
| --- | --- | --- | --- | --- |
| H1 |  | 3343 |  | 100 |
| H3 |  | 3202 |  | 100 |
| H4 |  | 896 |  | 99.67 |
| H5 |  | 2343 |  | 100 |
| H6 |  | 1011 |  | 100 |
| H7 |  | 2200 |  | 100 |
| H9 |  | 1231 |  | 99.76 |

^a^Sequences were derived from the GISAID EpiFlu Database and the NCBI Database, and were analyzed by using the MAFFT multiple sequence alignment program (version 7). The number of isolates analyzed includes human and avian influenza viruses in the database.
