# Supplementary material for: Viral RNA-binding ability conferred by SUMOylation at PB1 K612 of influenza A virus is essential for viral pathogenesis and transmission
Source: PLoS Pathog. 2021 Feb 11;17(2):e1009336. doi: 10.1371/journal.ppat.1009336 (PMC7904188; doi:10.1371/journal.ppat.1009336)
Supplement: S2 Table — (DOCX) [file ppat.1009336.s002.docx]

| Virus | Frequency of the indicated amino acid at position 612 of PB1 protein^a^ | |
| --- | --- | --- |
|  | K612 | R612 |
| WSN-PB1_K612R_ (H1N1) stock virus | 0%^b^ | 100% |
| VN/1180-PB1_K612R_ (H5N1) stock virus | 0% | 100% |
| AH/1-PB1_K612R_ (H7N9) stock virus | 0% | 100% |

^a^Viral RNAs isolated from the stock viruses were deep sequenced.

^b^Frequencies < 0.1% are denoted as 0%.
