# Supplementary material for: Viral RNA-binding ability conferred by SUMOylation at PB1 K612 of influenza A virus is essential for viral pathogenesis and transmission
Source: PLoS Pathog. 2021 Feb 11;17(2):e1009336. doi: 10.1371/journal.ppat.1009336 (PMC7904188; doi:10.1371/journal.ppat.1009336)
Supplement: S3 Table — (DOCX) [file ppat.1009336.s003.docx]

| Virus | Stock virus^a^ | | Animal pair | Viruses recovered from animals on different days post-inoculation (p.i.) or post-exposure (p.e.)^b^ | | | | | | | | | | | | | | | | | | | | |
| --- | --- | --- | --- | --- | --- | --- | --- | --- | --- | --- | --- | --- | --- | --- | --- | --- | --- | --- | --- | --- | --- | --- | --- | --- |
|  |  |  |  | Day 2 p.i. | |  | Day 4 p.i. | | |  | Day 3 p.e. | | |  | Day 5 p.e. | | | |  | | Day 7 p.e. | | |  |
|  | K612 | R612 |  | K612 | R612 |  | K612 | R612 | |  | K612 | R612 | |  | K612 | | R612 | |  | | K612 | | R612 |  |
| FZ/1-PB1_K612R_ (H1N1) | 0%^c^ | 100% | 1 | 98% | 2% |  | 100% | | 0% | | /^d^ | / |  | | / | / | |  | | / | | / | |  |
|  |  |  | 2 | 99% | 1% |  | 100% | | 0% | | / | / |  | | / | / | |  | | 99% | | 1% | |  |
|  |  |  | 3 | 31% | 69% |  | 100% | | 0% | | 1% | 99% |  | | 96% | 4% | |  | | 98% | | 2% | |  |

^a^Viral RNAs were isolated from the stock virus and results were obtained by deep sequencing.

^b^Viral RNAs were isolated from nasal washes and results were obtained by deep sequencing.

^c^Frequencies < 0.1% are denoted as 0%.

^d^Not applicable.
