# Supplementary figures and images for: Viral RNA-binding ability conferred by SUMOylation at PB1 K612 of influenza A virus is essential for viral pathogenesis and transmission
Source: PLoS Pathog. 2021 Feb 11;17(2):e1009336. doi: 10.1371/journal.ppat.1009336 (PMC7904188; doi:10.1371/journal.ppat.1009336)

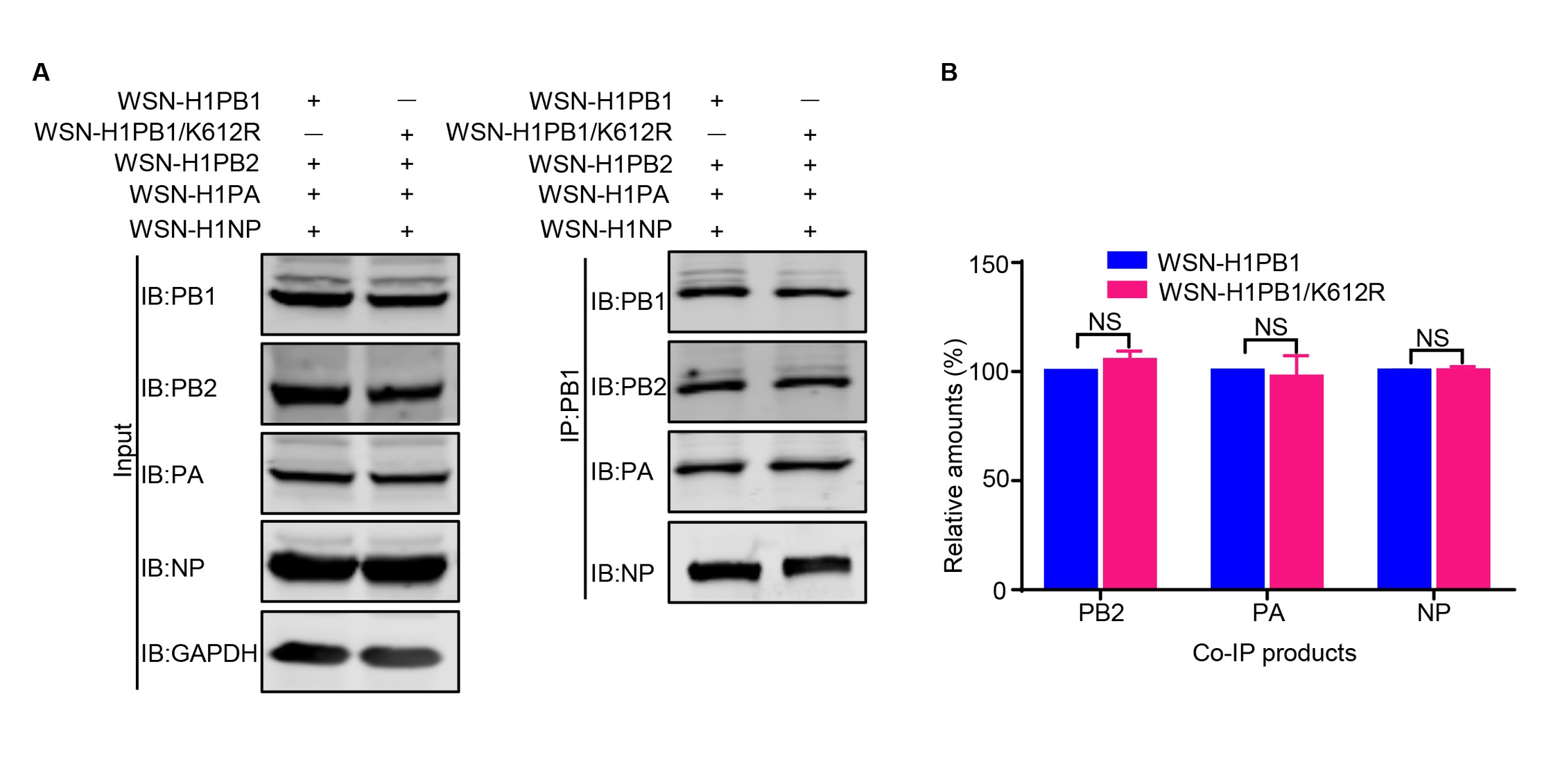

Supplement: S1 Fig — (A) HEK293T cells were cotransfected with plasmids encoding PB2, PB1 or PB1/K612R, PA, and NP of WSN (H1N1) virus, together with the pHH21-SC09NS F-Luc construct. Forty-eight hours later, cell lysates were immunoprecipitated with a mouse anti-PB1 mAb, followed by western blotting to detect the vRNP components, PB2, PA, and NP. (B) Quantification of immunoprecipitated PB2, PA, NP in (A) by using ImageJ software. The amounts of precipitated PB2, PA, and NP were normalized to the amount of precipitated PB1. The results are expressed as the mean ± SD of three assays and the significance was tested with a multiple t test (B). NS, not significant. (TIF) [file ppat.1009336.s004.tif]

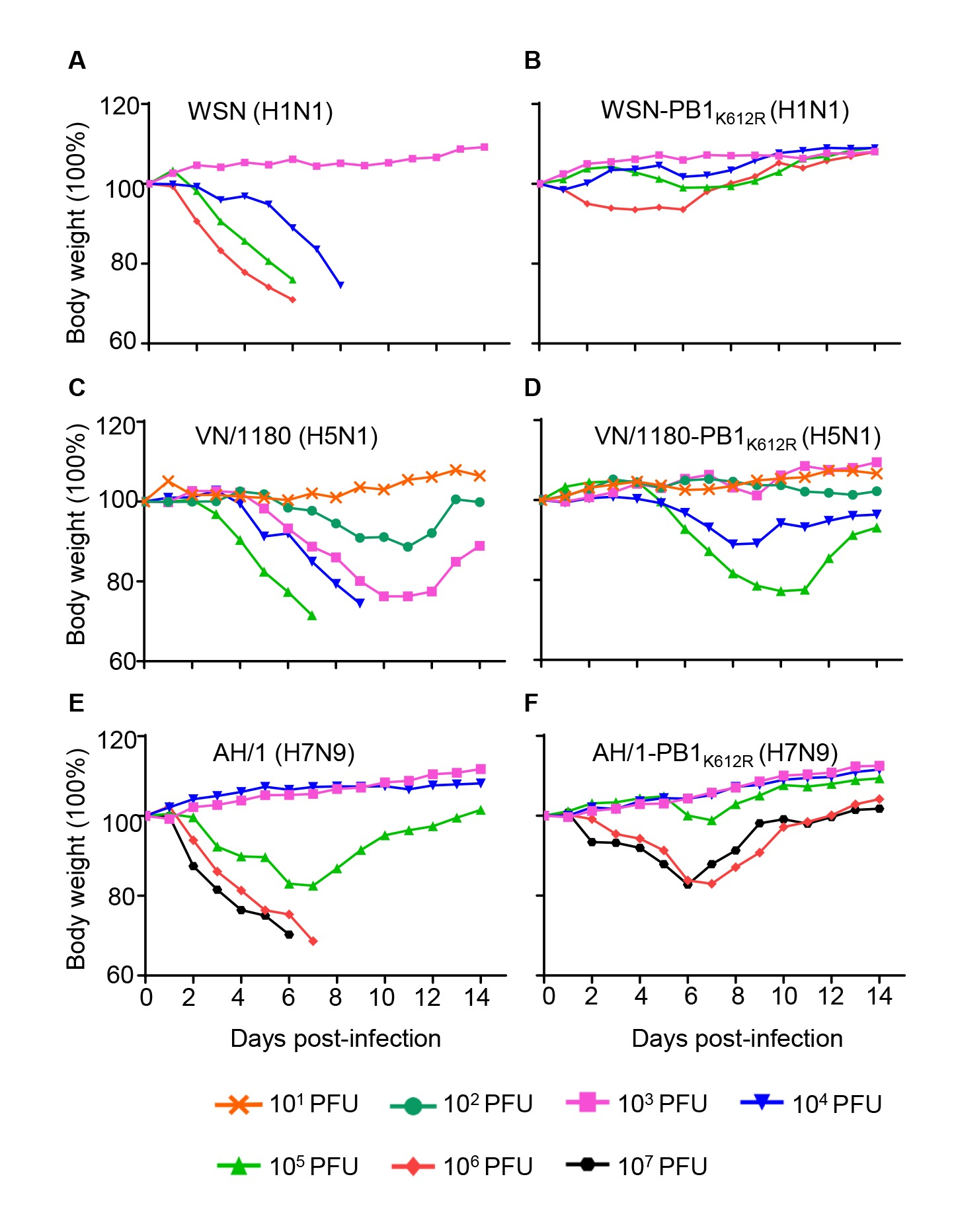

Supplement: S2 Fig — (A-F) Five mice per group were inoculated intranasally with the indicated doses of WSN (H1N1) (A), WSN-PB1K612R (H1N1) (B), VN/1180 (H5N1) (C), VN/1180-PB1K612R (H5N1) (D), AH/1 (H7N9) (E), or AH/1-PB1K612R (H7N9) (F) virus. Body weights were monitored daily for 14 days. (TIF) [file ppat.1009336.s005.tif]
